# Efficacy and Safety of Subcutaneous Vedolizumab in Patients With Moderately to Severely Active Crohn’s Disease: Results From the VISIBLE 2 Randomised Trial

**DOI:** 10.1093/ecco-jcc/jjab133

**Published:** 2021-08-17

**Authors:** Séverine Vermeire, Geert D’Haens, Filip Baert, Silvio Danese, Taku Kobayashi, Edward V Loftus, Siddharth Bhatia, Christian Agboton, Maria Rosario, Chunlin Chen, Wenwen Zhang, Krisztina Kisfalvi, William J Sandborn

**Affiliations:** 1 Department of Gastroenterology and Hepatology, University Hospitals Leuven, Leuven, Belgium; 2 Department of Gastroenterology, Amsterdam University Medical Centers, Amsterdam, The Netherlands; 3 Department of Gastroenterology, AZ Delta, Roeselare, Belgium; 4 Humanitas Clinical and Research Center – IRCCS, and Department of Biomedical Sciences, Humanitas University, Milan, Italy; 5 Center for Advanced IBD Research and Treatment, Kitasato University Kitasato Institute Hospital, Tokyo, Japan; 6 Division of Gastroenterology and Hepatology, Mayo Clinic College of Medicine, Rochester, MN, USA; 7 Takeda, Cambridge, MA, USA; 8 Takeda, Zurich, Switzerland; 9 Division of Gastroenterology, University of California San Diego, La Jolla, CA, USA

**Keywords:** Crohn’s disease, immunotherapy, subcutaneous

## Abstract

**Background and Aims:**

To report results from VISIBLE 2, a randomised, double-blind, placebo-controlled, phase 3 trial evaluating a new subcutaneous [SC] vedolizumab formulation as maintenance treatment in adults with moderately to severely active Crohn’s disease [CD].

**Methods:**

Following open-label vedolizumab 300 mg intravenous induction therapy at Weeks 0 and 2, Week 6 clinical responders (≥70-point decrease in CD Activity Index [CDAI] score from baseline) were randomised 2:1 to receive double-blind maintenance vedolizumab 108 mg SC or placebo every 2 weeks until Week 50. Assessments at Week 52 included clinical remission [primary endpoint; CDAI ≤150], enhanced clinical response [≥100-point decrease in CDAI from baseline], corticosteroid-free clinical remission among patients using a corticosteroid at baseline, clinical remission in anti-tumour necrosis factor [anti-TNF]-naïve patients, and safety.

**Results:**

Following vedolizumab intravenous induction, 275 patients were randomised to vedolizumab SC and 135 to placebo maintenance. At Week 52, 48.0% of patients receiving vedolizumab SC versus 34.3% receiving placebo were in clinical remission [*p* = 0.008]. Enhanced clinical response at Week 52 was achieved by 52.0% versus 44.8% of patients receiving vedolizumab SC versus placebo, respectively [*p* = 0.167]. At Week 52, 45.3% and 18.2% of patients receiving vedolizumab SC and placebo, respectively, were in corticosteroid-free clinical remission, and 48.6% of anti-TNF-naïve patients receiving vedolizumab SC and 42.9% receiving placebo were in clinical remission. Injection site reaction was the only new safety finding observed for vedolizumab SC [2.9%].

**Conclusions:**

Vedolizumab SC is an effective and safe maintenance therapy in patients with CD who responded to two infusions of vedolizumab intravenous induction therapy.

## Graphical Abstract

**Graphical Abstract:**
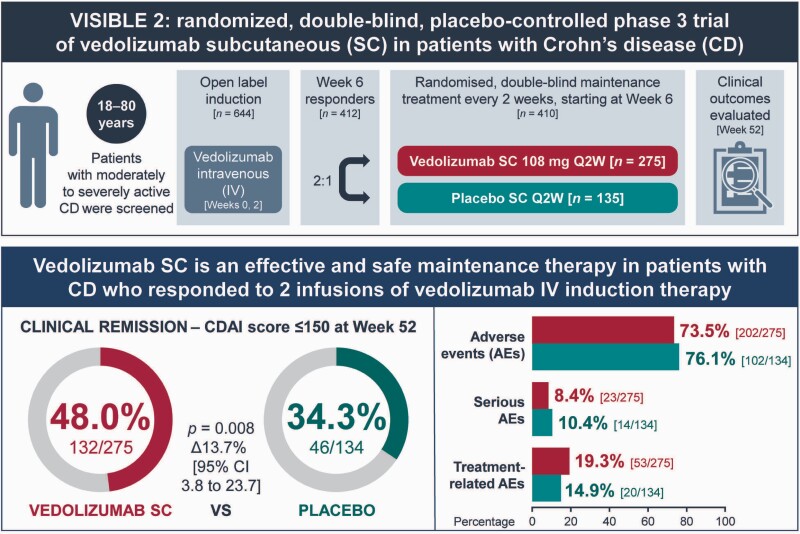

## 1. Introduction

Crohn’s disease [CD] is an inflammatory bowel disorder characterised by abdominal pain, diarrhoea, fatigue, and weight loss.^1,2^ When inadequately controlled, it can lead to structural bowel damage with stricture and/or penetrating disease and loss of function, negatively affecting quality of life [QoL] and work productivity.^3^ Vedolizumab, an anti-α _4_β _7_ integrin that selectively blocks lymphocyte trafficking to the gut, is approved worldwide as an intravenous [IV] formulation to treat moderately to severely active ulcerative colitis [UC] and CD.^4–7^ The efficacy and safety of vedolizumab IV 300 mg as both induction and maintenance therapy is well established.^8–10^

Most advanced treatments for moderately to severely active UC and CD are administered as IV infusions or subcutaneous [SC] injections.^11,12^ Patients may view an SC formulation as less time consuming and more convenient,^13,14^ especially for maintenance therapy. An SC formulation of vedolizumab [vedolizumab SC] was developed to provide an alternative route of vedolizumab administration and was approved in 2020 for use in UC and CD in Europe, Canada, and Australia as maintenance therapy (108 mg every 2 weeks [Q2W]).^7,15,16^ Vedolizumab SC was clinically evaluated in patients with moderately to severely active UC and CD. Results from the phase 3 VISIBLE 1 trial in UC have been reported.^17^ Significantly higher rates of clinical remission [defined as a total Mayo score ≤2 and no subscore >1] and endoscopic improvement were observed with vedolizumab SC maintenance therapy compared with placebo at Week 52 in patients with UC who had responded to vedolizumab IV induction.^17^ Moreover, the efficacy and safety profiles of vedolizumab SC maintenance were comparable to those of the vedolizumab IV reference arm.^17^ Here, we report efficacy and safety results from the phase 3 VISIBLE 2 trial evaluating vedolizumab SC maintenance treatment in patients with CD.

## 2. Methods

As part of the VISIBLE 2 study, all patients provided written informed consent, and the trial was approved by the institutional review board of each participating institution.

### 2.1. Study population

Adults aged 18–80 years with moderately to severely active CD diagnosed ≥3 months before study enrolment, who had previously demonstrated an inadequate response to or intolerance of corticosteroids [CS], immunomodulators, and/or anti-tumour necrosis factor [TNF] therapies, were eligible; see Supplementary Table 1, available as Supplementary data at *ECCO-JCC* online, for complete trial inclusion and exclusion criteria.

### 2.2. Study design

VISIBLE 2 [NCT02611817; EudraCT 2015-000481-58] was a randomised, double-blind, placebo-controlled, phase 3 trial of vedolizumab SC as maintenance treatment in adults with moderately to severely active CD [Supplementary Figure 1, available as Supplementary data at *ECCO-JCC* online]. The study was conducted between December 2015 and May 2019. Patients were enrolled at 169 sites in 30 countries. After a 28-day screening period, all enrolled patients received open-label vedolizumab 300 mg IV at Weeks 0 and 2. Clinical response (defined as a ≥70-point decrease in CD Activity Index [CDAI] from baseline) was assessed at Week 6. Patients who responded to vedolizumab 300 mg IV induction at Week 6 were randomised 2:1 to maintenance vedolizumab 108 mg SC or to placebo, every 2 weeks [Q2W] beginning at Week 6 and continuing through Week 50. The vedolizumab SC dose was selected to provide comparable drug exposures to 300 mg vedolizumab IV every 8 weeks [Q8W] based on average serum concentrations at steady state.^17^ Patient randomisation was stratified by three factors: concomitant use of oral CS, clinical remission status [defined as CDAI score ≤150] at Week 6, and previous treatment failure with or exposure to anti-TNF therapy or concomitant immunomodulator [azathioprine, 6-mercaptopurine, or methotrexate] use. The proportion of patients who had previous exposure to, but not treatment failure on, an anti-TNF was limited to 10%. For patients receiving CS at baseline, CS tapering was mandatory during the maintenance treatment phase of the study. Patients who had recurrence of symptoms could escalate once, up to a maximum of their baseline CS dose, on the condition that tapering was re-initiated within 2 weeks. Patients who failed to taper CS, and required consistent high doses of CS, were discontinued from the trial; see Supplementary Methods, available as Supplementary data at *ECCO-JCC* online, for more information.

### 2.3. Study endpoints and assessments

#### 2.3.1. Efficacy

The primary endpoint was clinical remission [defined as CDAI score ≤150] at Week 52. Rank-ordered secondary endpoints were: enhanced clinical response (defined as a ≥100 decline in CDAI score from baseline [Week 0]) at Week 52; CS-free clinical remission [patients using oral CS at baseline who discontinued CS and were in clinical remission at Week 52]; and clinical remission at Week 52 in anti-TNF-naïve patients. Patient-reported clinical remission at Week 52 was assessed as exploratory efficacy endpoints according to three definitions based on CDAI diary items: two-item [abdominal pain and stool frequency subscores] patient-reported outcome [PRO2] score ≤8; three-item [abdominal pain, stool frequency, and general well-being subscores] PRO [PRO3] score ≤13; and mean daily stool frequency ≤1.5 with abdominal pain ≤1.^18^ Clinical remission cut-offs for PRO2 and PRO3 were chosen to correspond with CDAI <150, and the third definition corresponded with two of the three optimal cut points for CDAI remission.^18^

Exploratory efficacy endpoints also included changes in inflammation biomarkers of CD activity, including faecal calprotectin and C-reactive protein [CRP] assessed using stool and blood samples, respectively, collected at screening and Weeks 0 [CRP only], 6, 30, and 52. Some patients who enrolled at select sites volunteered to undergo ileocolonoscopies at screening and at the Week 52/early termination visit; endoscopic response and endoscopic remission were assessed based on the Simple Endoscopic Score for CD.

Lack of efficacy was defined as disease worsening [≥100 point increase in CDAI score from the Week 6 value on two consecutive visits and a minimum CDAI score of 220 points], need for rescue medication, or need for surgery. Any new medication or escalation of dose above baseline dose [except for anti-diarrhoeals] was considered a rescue medication. In regard to CS, an increase back to baseline dose in patients undergoing tapering was not considered rescue medication. Patients who discontinued the study due to lack of efficacy and showed disease worsening on or after Week 6, or those who received rescue medication beyond Week 14, were eligible to enter an open-label extension [OLE; NCT02620046] study to receive vedolizumab SC after completion of the Week 52/early termination trial assessments.^19^ These patients were also eligible for dose escalation in the OLE study from Q2W to weekly dosing of vedolizumab SC. Patients who withdrew from the study and did not participate in OLE were managed outside the study.

#### 2.3.2. Health-related QoL and work productivity

Patients completed validated instruments to measure QoL and work productivity at Weeks 0, 6, 30, and 52, including the Inflammatory Bowel Disease Questionnaire,^20^ the EuroQol 5-Dimensions visual analogue scale, and the Work Productivity and Activity Impairment–CD scale; see Supplementary Methods for more information.

#### 2.3.3. Safety/tolerability

Safety assessments included all adverse events [AEs], AEs of special interest, serious AEs, vital signs, results of standard laboratory clinical chemistry, haematology, coagulation and urinalysis tests, and 12-lead electrocardiogram results. All AEs, regardless of causality, were reported and monitored from study enrolment. All AEs were coded using the Medical Dictionary for Regulatory Activities. Pre-defined AEs of special interest included serious infections, progressive multifocal leukoencephalopathy [PML], liver injury, malignancies, infusion-related or injection site reactions, and systemic reactions/hypersensitivities.

#### 2.3.4. Pharmacokinetics and immunogenicity

Blood samples were drawn for determination of vedolizumab serum concentrations pre-dose at Weeks 0, 6, 8, 14, 22, 30, 38, 46, 50, and 68; at any time during the study visits at Weeks 7, 51, and 52; at any unscheduled visit due to disease exacerbation; and at the final safety follow-up visit. Vedolizumab serum concentrations were determined using a validated sandwich enzyme-linked immunosorbent assay, with a limit of quantification of 0.2 μg/ml.^21^ Vedolizumab anti-drug antibody [ADA] titres were assessed from blood samples collected at Weeks 0, 6, 8, 14, 22, 30, 38, 46, and 52, and at Week 68/final safety visit. Assessments of ADAs and neutralising ADAs were determined using validated drug-tolerant [≥50 µg/ml at 500 ng/ml positive control] electrochemiluminescence assays.^22^

### 2.4. Statistical analyses

#### 2.4.1. Overview

Efficacy was assessed in the full analysis set, which included all randomised patients who received at least one dose of placebo or vedolizumab as maintenance therapy. Statistical analyses were performed using version 9.4 of the SAS software [Cary, NC, USA]. Other than the biomarker endpoints, where analysis was based on observed data, missing data for continuous endpoints were imputed using the last available post-baseline observation carried forward method. Missing data for proportion-based endpoints used non-responder imputation, in which any patient with missing information for determination of endpoint status was considered as a treatment failure/non-responder in the analysis. All confidence intervals [CIs], statistical tests, and resulting *p*-values were reported as two-sided and assessed at α = 0.05 significance level. As a sensitivity analysis, primary and secondary endpoints were also analysed in the per-protocol set, which included all patients who did not violate the terms of the protocol in a way that would significantly impact on the study. All safety analyses were performed by treatment arm in the safety analysis set, which included all patients who received at least one dose of maintenance SC drug; incidence rates were summarised by treatment arm and no statistical comparisons were made.

#### 2.4.2. Sample size calculation

Assuming a clinical remission rate of 38% for vedolizumab and 22% for placebo at Week 52 after maintenance treatment, a sample size of 258 patients in the vedolizumab arm and 129 patients in the placebo arm was determined to provide 90% power to detect a treatment effect at a two-sided 0.05 level of significance. To ensure a randomised sample size of 387 patients, assuming 47% of patients entering induction would achieve clinical response at Week 6, approximately 824 patients needed to enrol in the study.

#### 2.4.3. Primary and secondary efficacy analyses

Count, percentage, and associated 95% CI using the Clopper‐Pearson method were reported for each treatment arm. The *p*-value and point estimates of the treatment difference for efficacy endpoints were analysed using the Cochran‐Mantel‐Haenszel test, adjusted for randomisation stratification factors (concomitant use of oral CS [except for the analysis of CS-free remission], clinical remission status at Week 6, and previous anti-TNF therapy failure/exposure or concomitant immunomodulator use). To control the overall type I error rate for the comparison between vedolizumab SC and placebo arms for the primary and secondary endpoints, a fixed-sequence statistical testing approach was applied. Statistical testing of each endpoint proceeded according to the endpoint rank order only until an endpoint was not statistically significant [*p* <0.05]. The remaining endpoints were not formally tested and *p*-values were considered nominal. Exploratory analyses were performed on the primary and all secondary endpoints to evaluate the treatment effect across subpopulations, with point estimates of the absolute treatment difference based on crude estimates and associated 95% CIs reported for subpopulations with ≥10 patients in both treatment arms.

### 2.5. Study oversight

This study was overseen by the sponsor, Takeda, and conducted by contracted clinical investigators. Medical and clinical monitoring was conducted by the sponsor and its designated representatives. A data safety monitoring board, independent of the sponsor, regularly reviewed unblinded safety data. An independent adjudication committee was established to review and adjudicate potential PML events. The clinical study protocol and all applicable protocol amendments, the investigator’s brochure, a sample informed consent form, and other study-related documents were reviewed and approved by the local or central institutional review boards of all study sites. This study was conducted in compliance with the informed consent regulations stated in the Declaration of Helsinki, International Conference on Harmonisation Guidelines for Good Clinical Practice, and all applicable local laws and regulations.

### 2.6. Patient and public involvement

Patients were not involved in the design, recruitment, conducting, or dissemination of the results of the study.

### 2.7. Data availability

The datasets, including the redacted study protocol, redacted statistical analysis plan, and individual participants’ data supporting the results reported in this article, will be made available within 3 months from initial request, to researchers who provide a methodologically sound proposal. The data will be provided after de-identification, in compliance with applicable privacy laws, data protection, and requirements for consent and anonymisation. Data are available upon request via application at [https://search.vivli.org].

## 3. Results

### 3.1. Study population

Of the 644 patients who received vedolizumab IV induction therapy, 412 [64%] achieved a clinical response at Week 6. Twenty patients who were later determined to have met the criteria for clinical response were not randomised, and 18 patients [four in the placebo arm and 14 in the vedolizumab SC arm] who did not meet the CDAI threshold of change for clinical response were randomised. A total of 410 patients were randomised at Week 6 to vedolizumab SC [*n* = 275] or placebo [*n* = 135] maintenance therapy [Supplementary Figure 2, available as Supplementary data at *ECCO-JCC* online]. One patient randomised to the placebo arm did not receive the allocated intervention. A total of 107 patients in the vedolizumab SC arm and 61 patients in the placebo arm prematurely discontinued the study drug [Supplementary Figure 2]. The main reason for discontinuation in both arms was lack of efficacy [vedolizumab SC, *n =* 78; placebo, *n =* 43].

Baseline patient demographics were generally balanced between the two treatment arms [Table 1]. There were some differences in disease characteristics. More patients receiving vedolizumab SC versus placebo had ileum-only disease presentation [24.0% vs 15.7%] at the time of enrolment. Over half of the patients had previous exposure to an anti-TNF therapy, with more receiving vedolizumab SC [61.1%] than placebo [53.0%]. Approximately one-third of patients in each arm received concomitant CS at the time of enrolment. Most patients had moderate disease [defined as a CDAI score ≤330] at baseline [Week 0].

**Table 1.** Patient demographics and baseline characteristics.

Parameter	Placebo [*n =* 134]	Vedolizumab SC [*n =* 275]
Age [years], mean [SD]	36.1 [12.9]	38.2 [13.9]
Male, *n* [%]	66 [49.3]	157 [57.1]
White, *n* [%]	124 [92.5]	250 [90.9]
Body weight [kg], mean [SD]	69.8 [18.1]	74.1 [19.0]
Current smoker, *n* [%]	26 [19.4]	54 [19.6]
Duration of CD [years], mean [SD]	8.2 [8.4]	9.5 [8.3]
Disease activity, *n* [%]		
Moderate [CDAI ≤330]	81 [60.4]	160 [58.2]
Severe [CDAI >330]	53 [39.6]	115 [41.8]
CDAI score, median [minimum to maximum]		
Baseline	309.0 [198.0 to 461.0]	318.0 [206.0 to 559.0]
Week 6^a^	147.5 [-3.0 to 326.0]	150.5 [-8.0 to 362.0]
Faecal calprotectin [µg/g], median [minimum to maximum]	870.5 [10 to 15 050]	736.0 [10 to 14 570]
Faecal calprotectin,^b^*n* [%]		
≤250 µg/g	25 [18.7]	51 [18.5]
>250 to ≤500 µg/g	22 [16.4]	49 [17.8]
>500 µg/g	85 [63.4]	174 [63.3]
CRP, *n* [%]		
≤2.87 mg/l	32 [23.9]	72 [26.2]
>2.87 to ≤5 mg/l	22 [16.4]	35 [12.7]
>5 to ≤10 mg/l	21 [15.7]	65 [23.6]
>10 mg/l	59 [44.0]	103 [37.5]
Disease location, *n* [%]		
Ileum only	21 [15.7]	66 [24.0]
Colon only	26 [19.4]	55 [20.0]
Ileocolonic	74 [55.2]	122 [44.4]
Other	13 [9.7]	31 [11.3]
Prior surgery for CD, *n* [%]	34 [25.4]	76 [27.6]
Anti-TNF naïve, *n* [%]	64 [47.8]	110 [40.0]
Prior anti-TNF use, *n* [%]	71 [53.0]	168 [61.1]
Prior use of IMM [only], *n* [%]	4 [3.0]	16 [5.8]
Prior use of oral CS [only], *n* [%]	23 [17.2]	67 [24.4]
Prior use of oral CS and IMM, *n* [%]	103 [76.9]	189 [68.7]
Concomitant medications, *n* [%]		
Only IMM	34 [25.4]	51 [18.5]
Only CS	31 [23.1]	64 [23.3]
IMM and CS	13 [9.7]	31 [11.3]
History of fistulising disease, *n* [%]	34 [25.4]	53 [19.3]
Draining fistula at baseline, *n* [%]	12 [9.0]	14 [5.1]
Extraintestinal manifestations, *n* [%]	84 [62.7]	157 [57.1]

Anti-TNF, anti-tumour necrosis factor; CD, Crohn’s disease; CDAI, Crohn’s Disease Activity Index; CRP, C-reactive protein; SD, standard deviation; IMM, immunoodulator; CS, corticosteroids; SC, subcutaneous.

^a^Data missing for one patient in the vedolizumab SC group.

^b^Data missing for two patients in the placebo group and one patient in the vedolizumab SC group.

### 3.2. Efficacy

#### 3.2.1. Clinical efficacy outcomes

**Figure 1.**
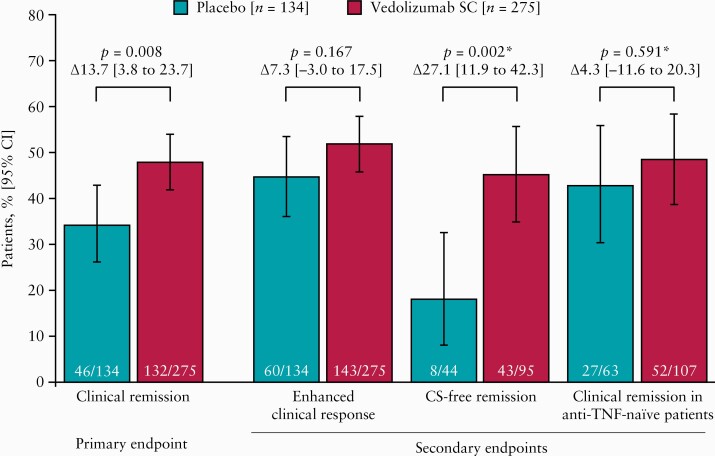
Primary and secondary endpoints at Week 52 [full analysis set]. The 95% CIs of the percentages for each treatment arm are based on the Clopper‐Pearson method. Treatment differences, the associated 95% CIs, and *p*-values are based on the Cochran‐Mantel‐Haenszel method, adjusted for randomisation strata. Patients missing data needed for the derivation of the endpoint are categorised as non-remitters or non-responders. CS use rates by clinical outcome are presented in Supplementary Table 7. *Nominal *p*-values that cannot be considered for statistical significance. Anti-TNF, anti-tumour necrosis factor; CI, confidence interval; CS, corticosteroids; SC, subcutaneous.

Of the randomised treated patients, 50.6% were in clinical remission and 84.4% showed enhanced clinical response at Week 6. At Week 52, significantly more patients receiving vedolizumab SC (132 of 275 [48.0%]) than placebo (46 of 134 [34.3%]) as maintenance treatment for CD were in clinical remission [∆13.7%; 95% CI 3.8 to 23.7%; *p =* 0.008] [Figure 1]. Enhanced clinical response at Week 52 was achieved by 143 of 275 [52.0%] and 60 of 134 [44.8%] patients receiving vedolizumab SC versus placebo, respectively [*p =* 0.167] [Figure 1]. CS-free clinical remission at Week 52 was achieved by 43 of 95 [45.3%] patients in the vedolizumab SC arm versus eight of 44 [18.2%] in the placebo arm [nominal *p =* 0.002], although statistical significance cannot be claimed due to lack of significance for enhanced clinical response [Figure 1]. Of anti-TNF-naïve patients, 52 of 107 [48.6%] versus 27 of 63 [42.9%] in the vedolizumab SC and placebo arms, respectively, were in clinical remission at Week 52 [nominal *p =* 0.591] [Figure 1]. The results of the primary and secondary endpoints analysed in the per-protocol set and in a post hoc sensitivity analysis excluding the 18 patients who did not meet clinical response criteria, and who were randomised to maintenance therapy, were generally consistent with the results in the full analysis set [Supplementary Table 2, available as Supplementary data at *ECCO-JCC* online]. The estimated treatment difference for enhanced clinical response was 12.9% for the per-protocol set [nominal *p =* 0.021].

**Figure 2.**
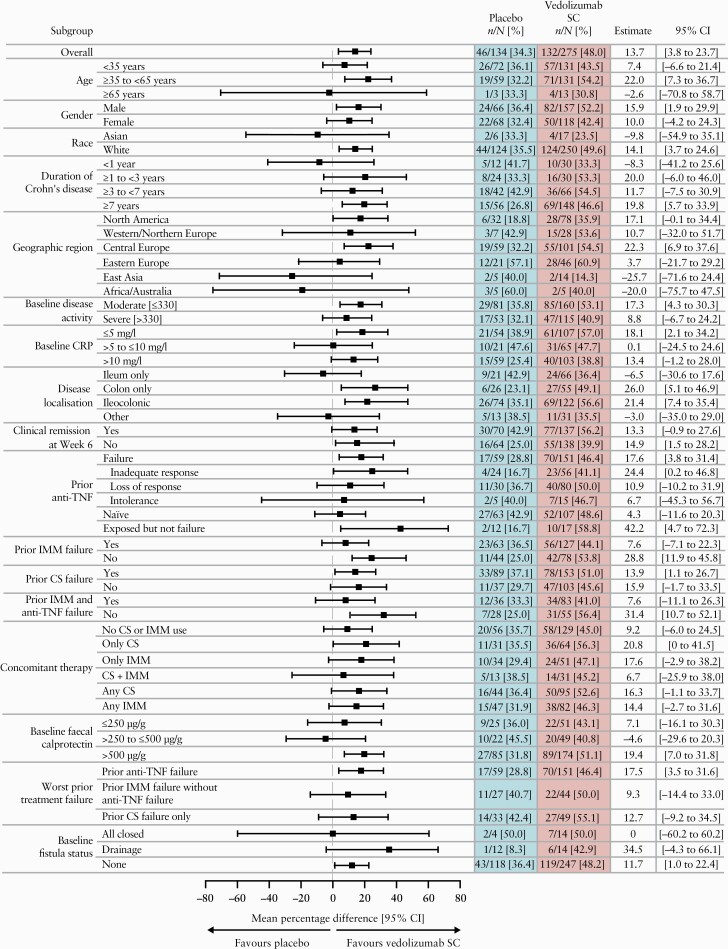
Clinical remission at Week 52 by subgroups based on key patient and disease characteristics [full analysis set]. Anti-TNF, anti-tumour necrosis factor; CI, confidence interval; CRP, C-reactive protein; CS, corticosteroids; IMM, immunomodulator; SC, subcutaneous.

Treatment differences in clinical remission at Week 52 were more pronounced in patients with previous anti-TNF failure, with 70 of 151 [46.4%] versus 17 of 59 [28.8%] anti-TNF-failure patients in the vedolizumab SC and placebo arms, respectively [nominal *p =* 0.019] [Figure 2]. Among anti-TNF-naïve patients, 16 of 39 [41.0%] receiving vedolizumab SC achieved CS-free clinical remission versus four of 22 [18.2%] receiving placebo. Among patients with previous anti-TNF failure, 24 of 52 [46.2%] versus three of 20 [15.0%] in the vedolizumab SC and placebo maintenance arms, respectively, achieved CS-free clinical remission. In a post hoc analysis, a larger proportion of anti-TNF-naïve patients randomised to vedolizumab SC had ileum-only disease (29 of 107 [27.1%]) compared with placebo [eight of 63 [12.7%]].

Treatment differences with clinical remission at Week 52 across a range of subgroups based on patient and disease characteristics were generally consistent with the overall population [Figure 2]. Notably, a treatment difference in clinical remission favouring vedolizumab SC over placebo was observed in patients with colonic or ileocolonic disease localisation, but not with ileum-only disease. Treatment differences with enhanced clinical response were generally consistent with the overall population, including in all anti-TNF subgroups [Supplementary Figure 3, available as Supplementary data at *ECCO-JCC* online].

**Figure 3.**
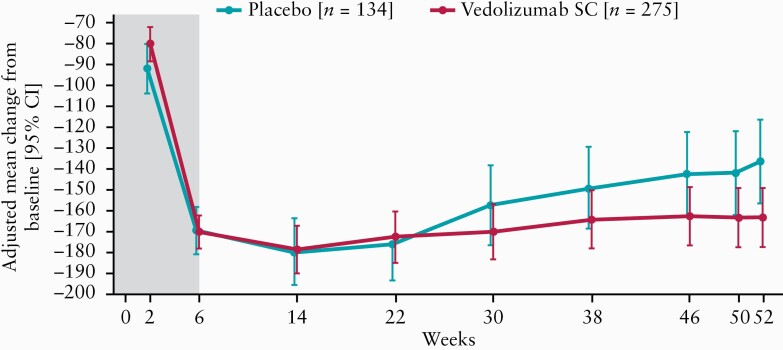
Change in Crohn’s Disease Activity Index scores by study visit [full analysis set]. Missing data were imputed using last available observation carried forward method. Least squares means and 95% CIs were obtained using an analysis of covariance model, with treatment as a factor and baseline score as a covariate at each visit. CI, confidence interval; SC, subcutaneous.

**Figure 4.**
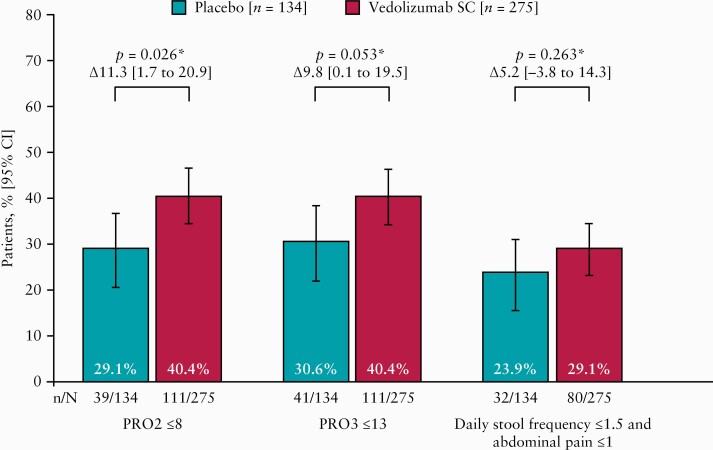
Clinical efficacy at Week 52 based on Crohn’s Disease Activity Index PROs, defined as a score ≤8 for PRO2 [abdominal pain and stool frequency subscores] and ≤13 for PRO3 [abdominal pain, stool frequency, and general well-being subscores]. *Nominal *p*-values that cannot be considered for statistical significance. CI, confidence interval; PRO, patient-reported outcome; SC, subcutaneous.

Patients receiving vedolizumab SC following vedolizumab IV induction showed greater improvements in CDAI scores over time compared with patients receiving placebo for maintenance [Figure 3]. Following vedolizumab IV induction, a higher proportion of patients on maintenance treatment with vedolizumab SC than placebo reported improvements in PRO2 and PRO3 [Figure 4]. The limited ileocolonoscopy data available from a subset of patients are presented in the Supplementary Results, available as Supplementary data at *ECCO-JCC* online.

#### 3.2.2. Biomarker endpoints

**Figure 5.**
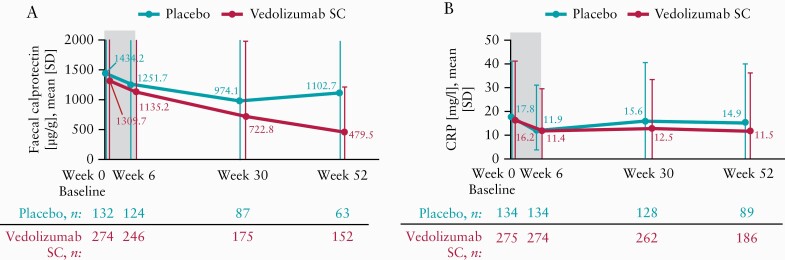
Observed [A] faecal calprotectin and [B] CRP by study visit [full analysis set]. CRP, C-reactive protein; SC, subcutaneous; SD, standard deviation.

There were improvements in faecal calprotectin and serum CRP concentrations over time [Figure 5]. Normal [≤250 µg/g] faecal calprotectin concentrations at Week 52 were detected in 60.5% versus 31.7% of patients in the vedolizumab SC versus placebo arms, respectively [Supplementary Figure 4 and Table 3, available as Supplementary data at *ECCO-JCC* online]. Among the patients in the vedolizumab SC and placebo arms, 61.1% [168 of 275] and 59.7% [80 of 134], respectively, had elevated CRP [>5 mg/l] at baseline. Of these patients, 23.2% in the vedolizumab SC arm and 17.5% in the placebo arm, had normalised CRP [≤5 mg/l] at Week 52 [Supplementary Figure 4, available as Supplementary data at *ECCO-JCC* online].

#### 3.2.3. Health-related QoL and work productivity

Early improvements in health-related QoL achieved during vedolizumab induction were maintained to a greater extent in patients receiving vedolizumab SC maintenance compared with placebo [Supplementary Figures 5–7, available as Supplementary data at *ECCO-JCC* online]. The difference between mean baseline and mean Week 52 total Inflammatory Bowel Disease Questionnaire scores was 48.7 points for patients receiving vedolizumab SC and 39.7 points for those receiving placebo.

#### 3.2.4. CS use at week 52

Among patients who were taking concomitant CS at baseline and achieved enhanced clinical response at Week 52, a post hoc analysis showed proportionally more patients had failed to taper CS in the placebo group: eight of 21 [38.1%] versus eight of 53 [15.1%] receiving vedolizumab SC at Week 52. Similar results were observed in anti-TNF-naïve patients with concomitant CS use at baseline who achieved clinical remission at Week 52, with seven of 11 [63.6%] patients receiving placebo and five of 21 [23.8%] patients receiving vedolizumab SC maintenance failing to taper CS at Week 52.

### 3.3. Safety/tolerability

Overall safety results were similar between the vedolizumab SC and placebo maintenance arms, with most AEs considered mild to moderate [Table 2]. A total of 22 patients discontinued the study drug due to AEs: 11 [4.0%] patients receiving vedolizumab SC and 11 [8.2%] receiving placebo.

**Table 2.** Overview of AEs [safety analysis set^a^].

Variable, *n* [%]	Placebo [*n =* 134]	Vedolizumab SC [*n =* 275]
AEs	102 [76.1]	202 [73.5]
Related	20 [14.9]	53 [19.3]
Not related	82 [61.2]	149 [54.2]
Mild	44 [32.8]	89 [32.4]
Moderate	46 [34.3]	99 [36.0]
Severe	12 [9.0]	14 [5.1]
Leading to study drug discontinuation	11 [8.2]	11 [4.0]
Serious AEs	14 [10.4]	23 [8.4]
Related	2 [1.5]	4 [1.5]
Not related	12 [9.0]	19 [6.9]
Leading to study drug discontinuation	5 [3.7]	5 [1.8]
Serious infections and infestations	6 [4.5]	4 [1.5]
Deaths	0	0

AE, adverse event; SC, subcutaneous.

^a^The safety analysis set included all patients who were randomised to the maintenance phase and received at least one dose of study drug.

The most frequently reported AEs were gastrointestinal disorders, including worsening of CD and abdominal pain [Table 3]. Nasopharyngitis and upper respiratory infections were more common with vedolizumab SC [9.1% and 6.2%, respectively] than placebo [4.5% and 3.7%, respectively]. Injection site reactions occurred in 2.9% of the vedolizumab SC arm versus 1.5% in the placebo arm [Supplementary Table 4, available as Supplementary data at *ECCO-JCC* online]. Overall, 37 [9.0%] patients experienced hypersensitivity-related AEs. Hypersensitivity-related AEs [which included Standardised Medical Dictionary for Regulatory Activities Queries for anaphylactic/anaphylactoid shock conditions, angioedema, and hypersensitivity], which were all mild or moderate, except for one case of seasonal allergy unrelated to vedolizumab SC, occurred at a rate of 8.7% in the vedolizumab SC arm versus 9.7% in the placebo arm. Malignancies were reported in two [0.7%] patients treated with vedolizumab SC and three [2.2%] treated with placebo.

**Table 3.** Most frequent [≥5% in any treatment arm] AEs by preferred term [safety analysis set^a^].

Variable, *n* [%]	Placebo [*n =* 134]	Vedolizumab SC [*n =* 275]
Patients with any most frequent AEs^b^	56 [41.8]	108 [39.3]
Crohn’s disease	26 [19.4]	42 [15.3]
Nasopharyngitis	6 [4.5]	25 [9.1]
Abdominal pain	11 [8.2]	21 [7.6]
Arthralgia	9 [6.7]	18 [6.5]
Upper respiratory infection	5 [3.7]	17 [6.2]
Headache	5 [3.7]	15 [5.5]
Nausea	7 [5.2]	11 [4.0]
Vomiting	7 [5.2]	6 [2.2]

Patients with one or more AE within a level of the Medical Dictionary for Regulatory Activities term were counted only once in that level.

AE, adverse event; SC, subcutaneous.

^a^The safety analysis set included all patients who were randomised to the maintenance phase and received at least one dose of study drug.

^b^Defined as an AE with date of onset occurring on or after the first dose of study drug in the induction period through 126 days after the latest dose date or before the first open-label extension dose, whichever occurred earlier.

Infections occurred in 86 [31.3%] patients receiving vedolizumab SC and 46 [34.3%] patients receiving placebo [Supplementary Table 5, available as Supplementary data at *ECCO-JCC* online]. Infection led to treatment discontinuation in two patients, both in the vedolizumab SC arm (one anal abscess [moderate severity] and one intestinal abscess [severe]). All infections classed as serious AEs [1.5% in vedolizumab SC; 4.5% in placebo] were moderate except for one severe case of appendicitis; all except one case of gastroenteritis were considered unrelated to study drug, and all patients fully recovered. Abdominal and gastrointestinal infections occurred in 11 [4.0%] patients receiving vedolizumab SC and seven [5.2%] patients receiving placebo. One patient [vedolizumab SC arm] developed a *Clostridium difficile* infection of moderate severity. No cases of PML and no deaths were reported.

### 3.4. Pharmacokinetics and immunogenicity

At Week 6, vedolizumab serum trough concentrations [C_trough_] were a median of 27.5 µg/ml [minimum to maximum, 0–76.7 µg/ml] in patients who switched to placebo maintenance following vedolizumab IV induction and 27.8 µg/ml [minimum to maximum, 0–68.1 µg/ml] in patients starting vedolizumab SC maintenance. Median vedolizumab C_trough_ at steady state [Week 46] in the placebo maintenance arm was 0 µg/ml [minimum to maximum, 0–31.9 µg/ml], whereas it was 30.2 µg/ml [minimum to maximum, 0.78–70.1 µg/ml] in the vedolizumab SC arm. A relationship between increasing vedolizumab exposure and the proportion of patients achieving clinical remission and enhanced clinical response was observed [Supplementary Figure 8, available as Supplementary data at *ECCO-JCC* online]. Vedolizumab ADAs were detected in seven of 275 [2.5%] patients receiving vedolizumab SC and 32 of 134 [23.9%] receiving placebo, following vedolizumab IV induction at Weeks 0 and 2 [Table 4].

**Table 4.** Summary of ADA status [safety analysis set^a^].

Overall ADA, *n* [%]	Placebo^b^ [*n =* 134]	Vedolizumab SC [*n =* 275]
ADA-negative^c^	102 [76.1]	268 [97.5]
ADA-positive^d^	32 [23.9]	7 [2.5]
Transiently positive^e^	8 [6.0]	4 [1.5]
Persistently positive^f^	24 [17.9]	3 [1.1]
Neutralising ADA^g^	18 [13.4]	4 [1.5]

All patients with missing data for determination of endpoint status were categorised as non-remitters. Overall ADA was defined from baseline [inclusive] through Week 52.

ADA, anti-drug antibody; SC, subcutaneous.

^a^The safety analysis set included all patients who were randomised to the maintenance phase and received at least one dose of study drug.

^b^Patients in the placebo arm received open-label vedolizumab during the 6-week induction phase but received placebo during the maintenance phase.

^c^Negative ADA was defined as a negative [not confirmed positive] ADA result at all visits.

^d^Positive ADA was defined as a confirmed ADA-positive result at one or more visits.

^e^Transiently positive ADA was defined as confirmed positive ADA result for at least one visit and no consecutive positive results.

^f^Persistently positive ADA was defined as a confirmed positive ADA result at two or more consecutive visits.

^g^Positive neutralising ADA was defined as a positive result in the neutralising ADA assay at any visit.

Among patients with samples available for ADA analysis, two of 132 patients in the placebo arm developed injection site reactions during maintenance treatment [both ADA-negative], and seven of 267 patients in the vedolizumab SC arm developed injection site reactions during maintenance treatment, of whom one was ADA-positive. Of patients with at least one ADA sample, hypersensitivity reactions during maintenance treatment occurred in 16 of 267 patients receiving vedolizumab SC [all ADA-negative] and 10 of 132 patients receiving placebo [one ADA-positive]. Of patients in clinical remission at Week 52, 13 of 46 [28.3%] were ADA-positive in the placebo arm and two of 132 [1.5%] were ADA-positive with vedolizumab SC [Supplementary Table 6, available as Supplementary data at *ECCO-JCC* online].

## 4. Discussion

VISIBLE 2 met its primary endpoint, demonstrating a significantly greater clinical remission rate at Week 52 for vedolizumab SC versus placebo in patients with moderately to severely active CD. This study followed the recently reported VISIBLE 1 clinical trial in which vedolizumab SC maintenance treatment was demonstrated to be effective and safe in patients with moderately to severely active UC. The treatment effect of vedolizumab SC maintenance therapy for clinical remission at Week 52 in CD patients has been consistent with that of the IV formulation observed in the GEMINI 2 study: clinical remission rates at Week 52 in the vedolizumab SC and placebo arms in VISIBLE 2 were 48.0% versus 34.3% [treatment difference 13.7%], and were 39.0% and 36.4% for vedolizumab IV Q8W and every 4 weeks [Q4W], respectively, versus 21.6% for placebo (treatment differences of 17.4% [Q8W] and 14.8% [Q4W]) in the GEMINI 2 trial.^8^

Treatment effects across the secondary efficacy endpoints consistently favoured vedolizumab SC over placebo in VISIBLE 2. The first-ranked secondary endpoint of enhanced clinical response, although not statistically significant, was higher with vedolizumab SC than placebo [treatment effect 7.3%]. In the next secondary endpoint, the proportion of patients achieving CS-free clinical remission at 52 weeks demonstrated a clinically meaningful treatment effect [27.1%] of vedolizumab SC over placebo; this comparison was not assessed for significance due to the pre-specified rank order analysis of secondary endpoints. The results for the final secondary endpoint of clinical remission in the anti-TNF-naïve population were similar between vedolizumab SC and placebo, with a small treatment difference [4.3%] for vedolizumab SC over placebo.

Higher rates of CDAI-based PRO2 and PRO3 clinical remission were observed with vedolizumab SC maintenance treatment compared with placebo, suggesting that vedolizumab SC may enhance relief of patient-perceived symptoms.

The limited treatment effects observed for vedolizumab SC versus placebo for some of the key endpoints, such as enhanced clinical response and clinical remission rates in anti-TNF-naïve patients in VISIBLE 2, are not fully understood, but higher placebo rates compared with GEMINI 2 may have an impact. Several factors might have, at least in part, contributed to the higher placebo rates observed in VISIBLE 2. First, differences in the VISIBLE 2 and GEMINI 2 study designs may have led to expectation bias: all patients in the VISIBLE 2 study received open-label vedolizumab IV induction, whereas GEMINI 2 used a double-blind, placebo-controlled, induction treatment phase. At Week 6 in the VISIBLE 2 study, clinical remission was observed in 50.6% of patients who were randomised to maintenance phase. Clinical efficacy following induction appears higher than that observed in the GEMINI 2 study.^8^ There were no noticeable differences in the baseline demographics and disease characteristics between patients with clinical response at Week 6 who were assigned to the placebo arm in the maintenance phase in the VISIBLE 2 and GEMINI 2 studies.^8^ In addition, there was 2:1 randomisation to vedolizumab SC or placebo in VISIBLE 2 compared with 1:1 to vedolizumab IV [Q8W] or placebo in GEMINI 2.^23,24^ Second, there may have been a potential confounding effect of CS at Week 52; all patients receiving CS at baseline were required to taper in the study, as described in Methods. In a post hoc analysis, more patients in the placebo group were still receiving oral CS at Week 52 compared with the vedolizumab SC group among those achieving enhanced clinical response at Week 52 [38.1% vs 15.1%] and those anti-TNF-naïve patients achieving clinical remission at Week 52 [63.6% vs 23.8%] [Supplementary Table 7, available as Supplementary data at *ECCO-JCC* online]. The contribution of CS to the overall clinical improvement observed in these patients is difficult to ascertain. Supporting the impact of concomitant CS use at Week 52 in the placebo group is the lower placebo rate [18.2% placebo vs 45.3% for vedolizumab SC] for the secondary endpoint of CS-free clinical remission, resulting in greater treatment effects observed for vedolizumab SC [27.1%]. Finally, a higher proportion of patients with ileum-only disease were randomised into the vedolizumab SC anti-TNF-naïve group compared with the placebo group (29 of 107 [27.1%] vs eight of 63 [12.7%]). It is well known that biologic therapies are more effective in patients with colon involvement than in those with ileum-only disease localisation.^25^ The relevance of this imbalance relates to evidence that patients with isolated ileal CD, as opposed to colonic CD, are significantly less likely to achieve a response or remission with the biologic intervention.^4^

Subgroup analyses according to TNF status showed treatment differences in favour of vedolizumab SC over placebo for key endpoint analyses at Week 52 in both anti-TNF-naïve and -failure subgroups, with differences in clinical remission more pronounced in patients with history of previous anti-TNF failure. Treatment differences in CS-free clinical remission were similar in anti-TNF-naïve and -failure patients.

The safety of vedolizumab SC is consistent with the known safety profile of vedolizumab IV therapy in patients with CD, with the exception of injection site reactions, which occurred in 2.9% [eight of 275] of patients in VISIBLE 2.^8,26^

The observed pharmacokinetic vedolizumab exposure after maintenance on the SC formulation in CD patients reported in VISIBLE 2 was comparable with the same treatment regimen in UC patients in VISIBLE 1.^17^ Immunogenicity rates in VISIBLE 2 were similar to previous reports.^8,10,27,28^

This study had several limitations. A vedolizumab IV reference arm was not included. Whereas comparable vedolizumab exposure and clinical efficacy with vedolizumab 300 mg IV Q8W and vedolizumab 108 mg SC Q2W maintenance is well established in UC patients,^17^ these results would have provided additional data specific to CD patients. Another limitation is that the results of endoscopic assessments were not essential for inclusion criteria, mirroring the design of the GEMINI 2 study, and endoscopic outcomes were assessed on voluntary basis. Based on comparable efficacy of vedolizumab SC to vedolizumab IV in the GEMINI 2 study, combined with the results of the VERSIFY trial evaluating vedolizumab IV, which showed that clinical remission/response was achieved as well as endoscopic improvements in patients with CD, it is reasonable to speculate of comparable clinical benefits with vedolizumab SC.^8,29^ These data represent efficacy and safety after 1 year of treatment. Additional data are being collected from this patient cohort during the ongoing VISIBLE open-label extension study [NCT02620046], to evaluate the long-term benefits of vedolizumab SC maintenance treatment.^19^

In conclusion, VISIBLE 2 trial results establish the efficacy and safety of vedolizumab SC as maintenance treatment for patients with moderately to severely active CD who responded to vedolizumab IV induction. Vedolizumab SC maintenance treatment of CD demonstrated clinically meaningful and statistically significant superiority over placebo for the primary endpoint of clinical remission at Week 52. In addition, the clinically meaningful treatment difference observed for the CS-free clinical remission endpoint supports the CS-sparing effect of vedolizumab SC as maintenance treatment in CD. Vedolizumab SC was well tolerated with no new safety signals observed, with the exception of injection site reactions. These results support vedolizumab SC as an important treatment option for patients who require maintenance therapy for CD. Vedolizumab is the first gut-targeted biological treatment for inflammatory bowel disease to offer the option of both IV and SC routes of administration for maintenance therapy.

## Funding

This work was supported by Takeda. WJS was supported in part by the NIDDK-funded San Diego Digestive Diseases Research Center [P30 DK120515]. Medical writing support was provided by Rezan Sahinkaya, PhD, of ProEd Communications, Inc., and Milena Wagner, PhD, of Excel Medical Affairs, funded by Takeda.

## Conflict of Interest

SV has received research support from AbbVie, Johnson & Johnson, Pfizer, and Takeda; lecture fees from AbbVie, Centocor, Ferring, Genentech/Roche, Hospira, Johnson & Johnson, Merck Sharp & Dohme, Pfizer, Takeda, and Tillotts; and consulting fees from AbbVie, Abivax, Celgene, Celltrion, Centocor, Ferring, Galapagos, Genentech/Roche, Gilead, GlaxoSmithKline, Hospira, Johnson & Johnson, Merck Sharp & Dohme, Mundipharma, Pfizer, ProDigest, Prometheus, Second Genome, Takeda, and Tillotts. GDH has received research support from AbbVie, Bühlmann Laboratories, Dr Falk Pharma, Ferring, GlaxoSmithKline, Johnson & Johnson, Medtronics, Merck Sharp & Dohme, Millennium/Takeda, PhotoPill, Prometheus, Robarts Clinical Trials, and Setpoint; lecture fees from AbbVie, Biogen, Dr Falk Pharma, Ferring, Giuliani, Johnson & Johnson, Merck Sharp & Dohme, Millennium/Takeda, Norgine, Otsuka, Shire, Tillotts, UCB, and Vifor; consulting fees from AbbVie, Ablynx, ActoGeniX, Amakem, Amgen, AM-Pharma, AstraZeneca, Biogen, Boehringer Ingelheim, Bristol Myers Squibb, Celgene/Receptos, Celltrion, Cosmo, Elan, enGene, Ferring, Galapagos, Gilead, GlaxoSmithKline, Hospira/Pfizer, Immunic, Johnson & Johnson, Lycera, Medimetrics, Medtronics, Merck Sharp & Dohme, Millennium/Takeda, Mitsubishi Pharma, Mundipharma, Novo Nordisk, Otsuka, PDL, Prometheus, Protagonist, Salix, Samsung Bioepis, Sandoz, Setpoint, Shire, Teva, TiGenix, Tillotts, TopiVert, UCB, Versant, and Vifor; directorship at Robarts Clinical Trials. FB has received grant/research support from AbbVie, Amgen, Janssen, and Takeda; honoraria from AbbVie, Amgen, Arena, Celgene, Ferring, Fresenius Kabi, Janssen, Merck Sharp & Dohme, Pfizer, and Takeda; and was on speaker bureaus for AbbVie, Ferring, Janssen, Merck Sharp & Dohme, Pfizer, and Takeda. SD has received lecture and consulting fees from AbbVie, Allergan, Biogen, Boehringer Ingelheim, Celgene, Celltrion, Ferring, Hospira, Johnson & Johnson, Merck, Merck Sharp & Dohme, Mundipharma, Pfizer, Sandoz, Takeda, TiGenix, UCB, and Vifor. TK has received consulting fees from AbbVie, Alfresa, CovidienÐ, Eli Lilly, Ferring, Janssen, Kyorin, Mochida, Nippon Kayaku, Pfizer, Takeda, and Thermo Fisher Scientific; lecture fees from AbbVie, Ajinomoto Bio-Pharma, Alfresa, Asahi Kasei Medical, Astellas, Celltrion, EA Pharma, Eisai, Gilead, Janssen, JIMRO, Kyorin, Mitsubishi Tanabe Pharma, Mochida, Nippon Kayaku, Takeda, and Zeria; and research support from AbbVie, Alfresa, Asahi Kasei Medical, EA Pharma, Kyorin, Mochida, Nippon Kayaku, Otsuka, Thermo Fisher Scientific, and Zeria. EVL Jr has received research support from AbbVie, Amgen, Bristol Myers Squibb, Celgene, Genentech, Gilead, Janssen, Pfizer, Receptos, Robarts Clinical Trials, Takeda, Theravance Biopharma, and UCB; consulting fees from AbbVie, Allergan, Amgen, Arena, Boehringer Ingelheim, Bristol Myers Squibb, Calibr, Celgene, Celltrion, Eli Lilly, Genentech, Gilead, Iterative Scopes, Janssen, Ono Pharma, Pfizer, Sun Pharma, Takeda, and UCB; is a shareholder of Exact Sciences; and served as a medical editor for Healio/SLACK. SB, WZ, and KK are employees of Millennium Pharmaceuticals, a Takeda group company and hold stock/stock options in Takeda. CA is an employee of Takeda and holds stock/stock options in Takeda. MR and CC were employees of Millennium Pharmaceuticals, a Takeda group company at the time this research was conducted. WJS has received research grants from AbbVie, Abivax, Arena Pharmaceuticals, Boehringer Ingelheim, Celgene, Genentech, Gilead Sciences, GlaxoSmithKline, Janssen, Lilly, Pfizer, Prometheus Biosciences, Seres Therapeutics, Shire, Takeda, and Theravance Biopharma; consulting fees from AbbVie, Abivax, AdMIRx, Alfasigma, Alimentiv [previously Robarts Clinical Trials, owned by Alimentiv Health Trust], Alivio Therapeutics, Allakos, Amgen, Applied Molecular Transport, Arena Pharmaceuticals, Bausch Health [Salix], Beigene, Bellatrix Pharmaceuticals, Boehringer Ingelheim, Boston Pharmaceuticals, Bristol Myers Squibb, Celgene, Celltrion, Cellularity, Cosmo Pharmaceuticals, Escalier Biosciences, Equillium, Forbion, Genentech/Roche, Gilead Sciences, Glenmark Pharmaceuticals, Gossamer Bio, Immunic [Vital Therapies], Index Pharmaceuticals, Intact Therapeutics, Janssen, Kyverna Therapeutics, Landos Biopharma, Lilly, Oppilan Pharma, Otsuka, Pandion Therapeutics, Pfizer, Progenity, Prometheus Biosciences, Protagonists Therapeutics, Provention Bio, Reistone Biopharma, Seres Therapeutics, Shanghai Pharma Biotherapeutics, Shire, Shoreline Biosciences, Sublimity Therapeutics, Surrozen, Takeda, Theravance Biopharma, Thetis Pharmaceuticals, Tillotts Pharma, UCB, Vendata Biosciences, Ventyx Biosciences, Vimalan Biosciences, Vivelix Pharmaceuticals, Vivreon Biosciences, and Zealand Pharma; and holds stock/stock options in Allakos, BeiGene, Gossamer Bio, Oppilan Pharma, Prometheus Biosciences, Progenity, Shoreline Biosciences, Ventyx Biosciences, Vimalan Biosciences, and Vivreon Biosciences; spouse of WJS reports consulting for Iveric Bio and Oppilan Pharma; and holds stock/stock options in Iveric Bio, Oppilan Pharma, Progenity, Prometheus Biosciences, Ventyx Biosciences, and Vimalan Biosciences; employee of Prometheus Biosciences.

## Author Contributions

SV, KK, and WJS were involved in the conception and design of the study; GDH, FB, SD, TK, and EVL Jr were involved in the acquisition of the data; SV, GDH, FB, SD, TK, EVL Jr, SB, CA, MR, CC, WZ, KK, and WJS analysed and interpreted the data, drafted the article, or critically revised for intellectual content, and approved the final version for submission. Conference presentations: Australian Gastroenterology Week [AGW], virtual congress, 2020; Digestive Disease Week [DDW], virtual congress, 2020; European Crohn’s and Colitis Organisation [ECCO], Vienna, Austria, 2020; United European Gastroenterology Week [UEGW] – 28th Annual Conference, virtual congress, 2020.

## Supplementary Material

jjab133_suppl_Supplementary_Materials
