# Supplementary material for: Efficacy and Safety of Subcutaneous Vedolizumab in Patients With Moderately to Severely Active Crohn’s Disease: Results From the VISIBLE 2 Randomised Trial
Source: J Crohns Colitis. 2021 Aug 17;16(1):27–38. doi: 10.1093/ecco-jcc/jjab133 (PMC8797168; doi:10.1093/ecco-jcc/jjab133)
Supplement: jjab133_suppl_Supplementary_Materials [file jjab133_suppl_supplementary_materials.pdf]

Supplementary Data

Supplementary Results

Efficacy based on ileocolonoscopy

In patients who underwent voluntary ileocolonoscopy, higher proportions of patients receiving vedolizumab subcutaneous maintenance [six of 16] than placebo [three of 13] showed clinical remission based on an alternative definition [daily abdominal pain score of ≤1, and ≤10 total liquid/very soft stools for 7 days before visit, with Simple Endoscopic Score for CD ≤4- and ≥2-point reduction from baseline with no subscore >1]. The same was true using an alternative definition of endoscopic remission [Simple Endoscopic Score for Crohn's disease subscore ≤2; seven of 16 vs three of 13, respectively].

Supplementary Methods

Study population

Patients could continue oral corticosteroids [CS; prednisone at a stable dose ≤30 mg/day, budesonide at a stable dose ≤9 mg/day or equivalent] if just initiated and on a stable dose for 4 weeks before first study drug dose, or for 2 weeks prior, if tapering. For prednisone doses >10 mg/day or equivalent, doses were reduced by 5 mg/week until reaching 10 mg/day; for doses ≤10 mg/day or equivalent, doses were reduced by 2.5 mg/week until discontinuation. Budesonide was tapered at a rate of 3 mg every 3 weeks. Patients who had recurrence of symptoms during CS tapering could increase the dose once, up to the original maximum dose of their CS at baseline [CS dose at the start of induction therapy]. In those patients, tapering had to be reinitiated within 2 weeks. Patients who failed to taper CS and required consistent higher doses of CS, were discontinued from the trial.

Study endpoints and assessments

Health-related quality of life [QoL] and work productivity

Patients completed validated instruments to measure QoL and work productivity at Weeks 0, 6, 30, and 52. Inflammatory Bowel Disease Questionnaire domains [bowel systems, emotional function, social function, and systemic function] were rated on a 7-point Likert scale, with higher scores equating to higher QoL. Patients assessed their overall health using the EuroQol 5-Dimensions visual analogue scale on a scale from 0 as the worst to 100 as the best possible health. The Work Productivity and Activity Impairment–Crohn's Disease scale includes four metrics: absenteeism [work missed due to health in the previous 7 days], presenteeism [impairment at work due to health in the previous 7 days], overall work productivity loss [a combination of absenteeism and presenteeism], and activity impairment [impairment in daily activities due to health in the previous 7 days].

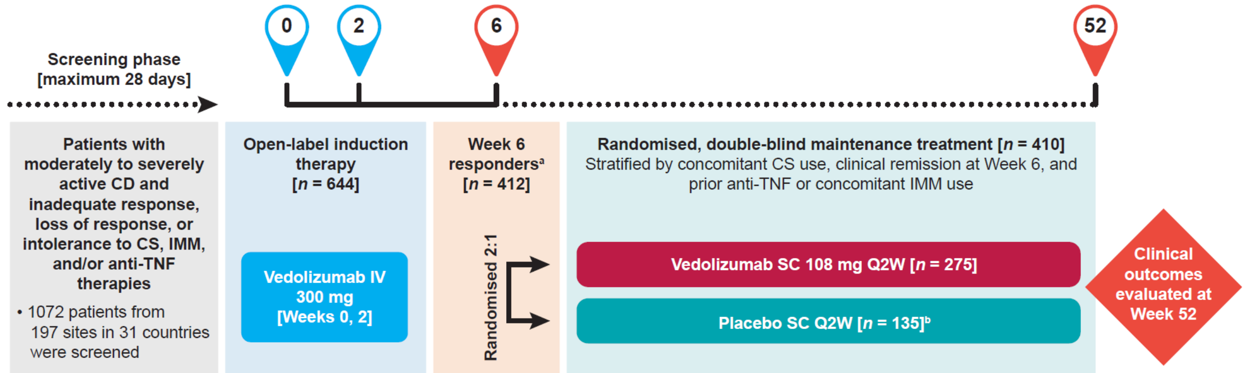

Supplementary Figure 1. Study design. [a]Week 6 clinical response is defined as a ≥70-point decrease in CD Activity Index from baseline. [b]One randomised patient did not receive the study drug. anti-TNF, anti-tumour necrosis factor; CD, Crohn's disease; CS, corticosteroids; IMM, immunomodulator; IV, intravenous; Q2W, every 2 weeks; SC, subcutaneous.

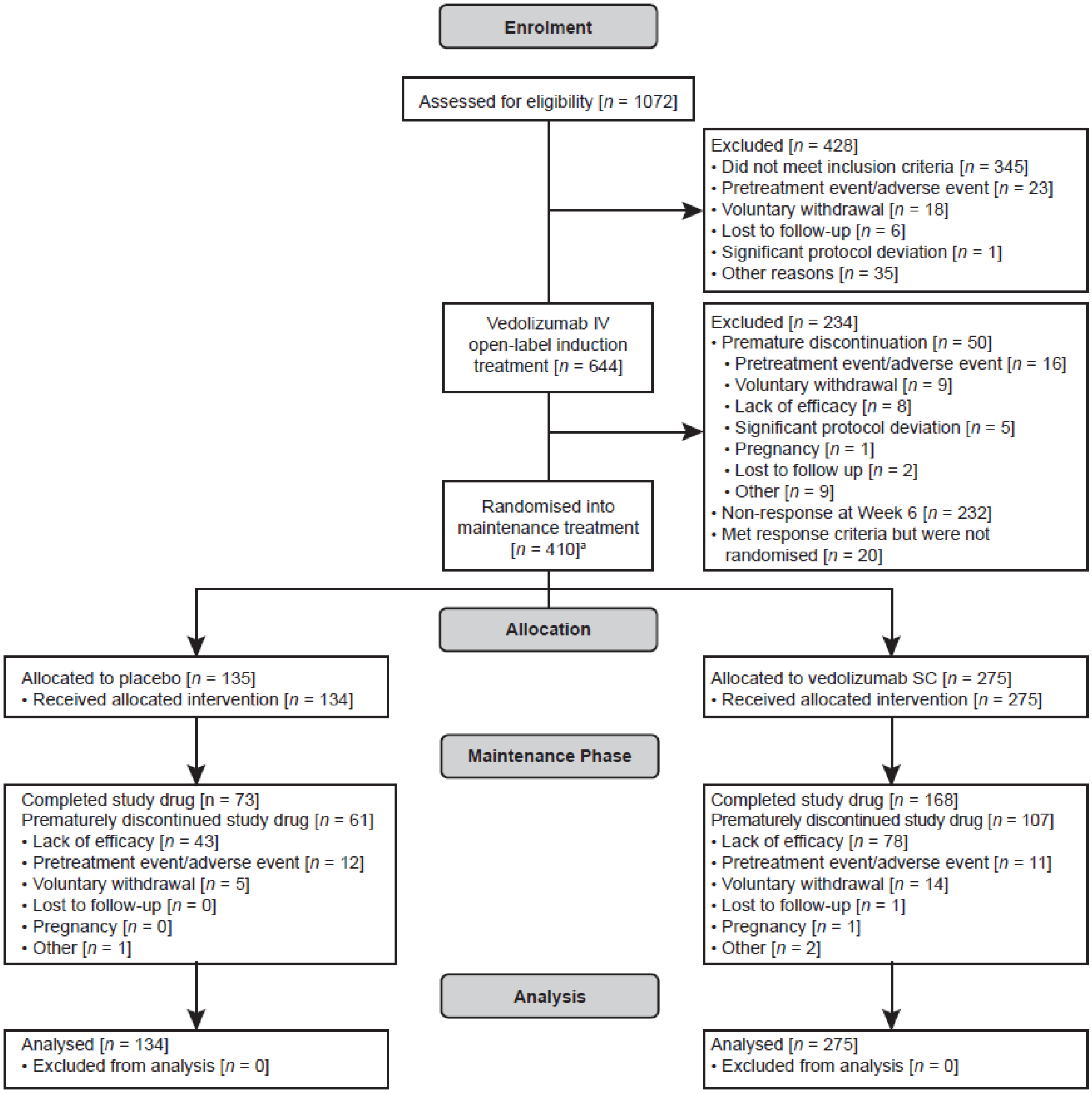

Supplementary Figure 2. Patient disposition. Randomisation procedure: randomisation personnel of the sponsor or designee generated the randomisation schedule before the start of the study. An interactive web response system was used for patient randomisation. All randomisation information was stored in a secured area, accessible only by authorised personnel. [a]Includes 18 patients who did not meet the criteria for response [≥70-point decrease in Crohn's Disease Activity Index score from baseline]. IV, intravenous; SC, subcutaneous.

Subgroup		Placebo n/N [%]	Vedolizumab SC n/N [%]	Estimate	95% CI
Overall		60/134 [44.8]	143/275 [52.0]	7.3	[-3.0 to 17.5]
Age	<35 years	36/72 [50.0]	63/131 [48.1]	-1.9	[-16.3 to 12.5]
	≥35 to <65 years	23/59 [39.0]	76/131 [58.0]	19.0	[4.0 to 34.1]
	≥65 years	1/3 [33.3]	4/13 [30.8]	-2.6	[-70.8 to 58.7]
Gender	Male	35/66 [53.0]	90/157 [57.3]	4.3	[-10.0 to 18.6]
	Female	25/68 [36.8]	53/118 [44.9]	8.2	[-6.4 to 22.7]
Race	Asian	3/6 [50.0]	5/17 [29.4]	-20.6	[-64.1 to 24.8]
	White	57/124 [46.0]	133/250 [53.2]	7.2	[-3.5 to 18.0]
Duration of Crohn's disease	<1 year	6/12 [50.0]	12/30 [40.0]	-10.0	[-43.3 to 23.3]
	≥1 to <3 years	9/24 [37.5]	16/30 [53.3]	15.8	[-10.5 to 42.2]
	≥3 to <7 years	21/42 [50.0]	38/66 [57.6]	7.6	[-11.7 to 26.8]
	≥7 years	24/56 [42.9]	76/148 [51.4]	8.5	[-6.8 to 23.8]
Geographic region	North America	8/32 [25.0]	31/78 [39.7]	14.7	[-0.1 to 34.4]
	Western/Northern Europe	4/7 [57.1]	15/28 [53.6]	-3.6	[-45.2 to 38.7]
	Central Europe	27/59 [45.8]	61/101 [60.4]	14.6	[-1.3 to 30.5]
	Eastern Europe	14/21 [66.7]	28/46 [60.9]	-5.8	[-30.4 to 18.8]
	East Asia	2/5 [40.0]	3/14 [21.4]	-18.6	[-65.7 to 31.0]
	Africa/Australia	3/5 [60.0]	3/5 [60.0]	0.0	[-62.6 to 62.6]
Baseline disease activity	Moderate [≤330]	32/81 [39.5]	86/160 [53.8]	14.2	[1.1 to 27.4]
	Severe [>330]	28/53 [52.8]	57/115 [49.6]	-3.3	[-19.5 to 13.0]
Baseline CRP	≤5 mg/l	24/54 [44.4]	69/107 [64.5]	20.0	[4.0 to 36.1]
	>5 to ≤10 mg/l	14/21 [66.7]	32/65 [49.2]	-17.4	[-41.0 to 6.1]
	≥10 mg/l	22/59 [37.3]	42/103 [40.8]	3.5	[-12.1 to 19.1]
Disease localisation	Ileum only	11/21 [52.4]	29/66 [43.9]	-8.4	[-32.9 to 16.0]
	Colon only	10/26 [38.5]	30/55 [54.5]	16.1	[-6.8 to 39.0]
	Ileocolonic	32/74 [43.2]	71/122 [58.2]	15.0	[0.7 to 29.2]
	Other	7/13 [53.8]	12/31 [38.7]	-15.1	[-47.2 to 16.9]
Clinical remission at Week 6	Yes	35/70 [50.0]	82/137 [59.9]	9.9	[-4.4 to 24.2]
	No	25/64 [39.1]	61/138 [44.2]	5.1	[-9.4 to 19.7]
Prior anti-TNF	Failure	27/59 [45.8]	74/151 [49.0]	3.2	[-11.8 to 18.2]
	Inadequate response	8/24 [33.3]	24/56 [42.9]	6.8	[-15.7 to 29.2]
	Loss of response	17/30 [56.7]	42/80 [52.5]	-7.2	[-28.6 to 14.1]
	Intolerance	2/5 [40.0]	8/15 [53.3]	13.3	[-39.3 to 62.5]
	Naïve	30/63 [47.6]	58/107 [54.2]	4.4	[-11.6 to 20.3]
	Exposed but not failure	3/12 [25.0]	11/17 [64.7]	39.7	[1.8 to 69.2]
Prior IMM failure	Yes	29/63 [46.0]	63/127 [49.6]	3.6	[-11.5 to 18.6]
	No	16/44 [36.4]	44/78 [56.4]	20.0	[2.1 to 38.0]
Prior CS failure	Yes	42/89 [47.2]	86/153 [56.2]	9.0	[-4.0 to 22.0]
	No	15/37 [40.5]	48/103 [46.6]	6.1	[-12.5 to 24.6]
Prior IMM and anti-TNF failure	Yes	19/36 [52.8]	36/83 [43.4]	-9.4	[-28.9 to 10.1]
	No	10/28 [35.7]	33/55 [60.0]	24.3	[2.3 to 46.3]
Concomitant therapy	No CS or IMM use	25/56 [44.6]	65/129 [50.4]	5.7	[-9.9 to 21.4]
	Only CS	15/31 [48.4]	38/64 [59.4]	11.0	[-10.3 to 32.3]
	Only IMM	14/34 [41.2]	25/51 [49.0]	7.8	[-13.6 to 29.3]
	CS + IMM	6/13 [46.2]	15/31 [48.4]	2.2	[-30.1 to 34.5]
	Any CS	21/44 [47.7]	53/95 [55.8]	8.1	[-9.8 to 25.9]
	Any IMM	20/47 [42.6]	40/82 [48.8]	6.2	[-11.6 to 24.0]
Baseline faecal calprotectin	≤250 µg/g	12/25 [48.0]	25/51 [49.0]	1.0	[-22.9 to 24.9]
	>250 to ≤500 µg/g	11/22 [50.0]	23/49 [46.9]	-3.1	[-28.2 to 22.1]
	>500 µg/g	37/85 [43.5]	94/174 [54.0]	10.5	[-2.4 to 23.4]
Worst prior treatment failure	Prior anti-TNF failure	27/59 [45.8]	74/151 [49.0]	3.2	[-11.8 to 18.2]
	Prior IMM failure without anti-TNF failure	10/27 [37.0]	27/44 [61.4]	24.3	[1.1 to 47.5]
	Prior CS failure only	18/33 [54.5]	28/49 [57.1]	2.6	[-19.3 to 24.5]
Baseline fistula status	All closed	2/4 [50.0]	7/14 [50.0]	0	[-6.2 to 60.2]
	Drainage	3/12 [25.0]	7/14 [50.0]	25.0	[-14.0 to 59.5]
	None	55/118 [46.6]	129/247 [52.2]	5.6	[-5.3 to 16.6]

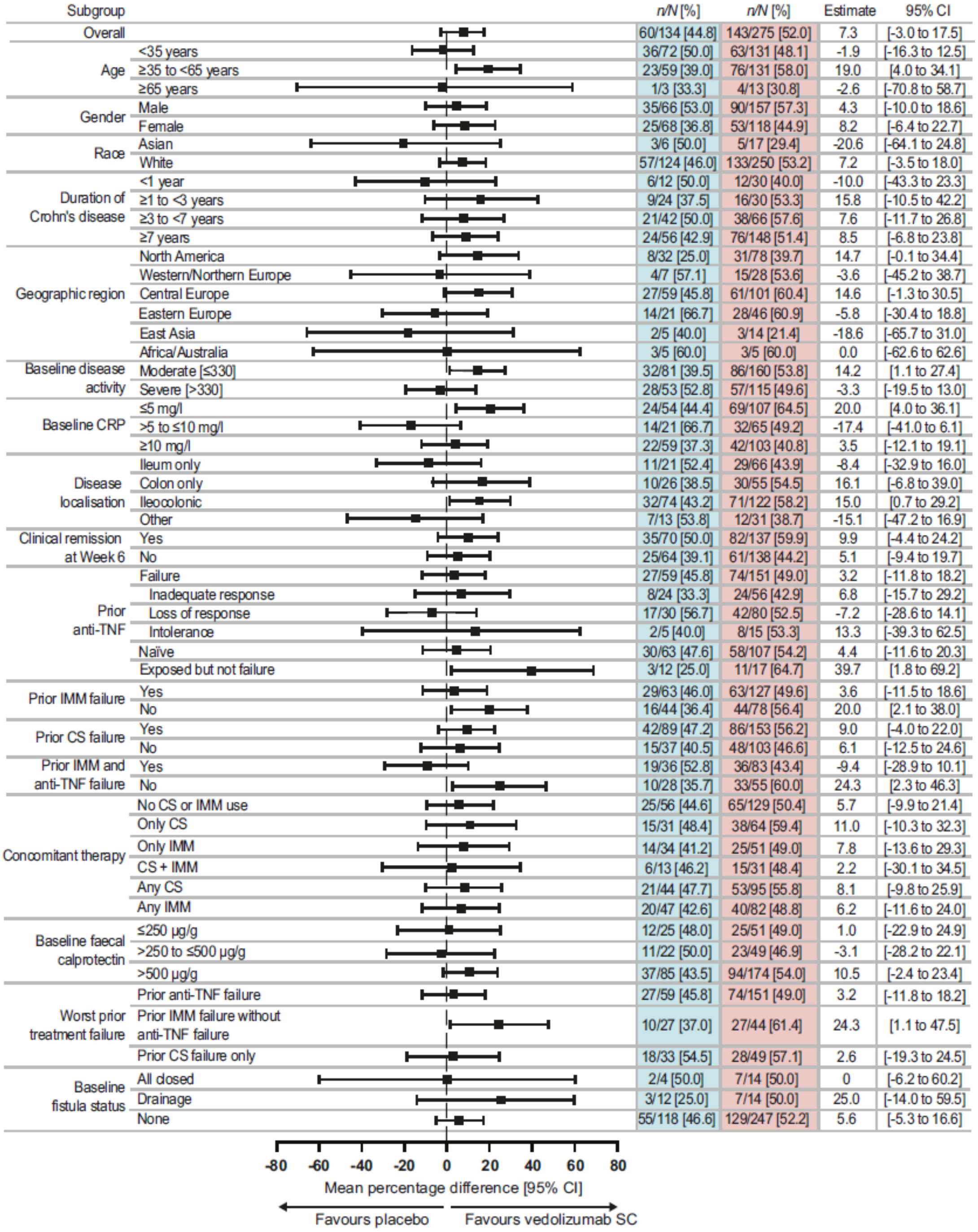

Supplementary Figure 3. Enhanced clinical response at Week 52 by subgroups based on key patient and disease characteristics [full analysis set]. anti-TNF, anti-tumour necrosis factor; CI, confidence interval; CRP, C-reactive protein; CS, corticosteroids; IMM, immunomodulator; SC, subcutaneous.

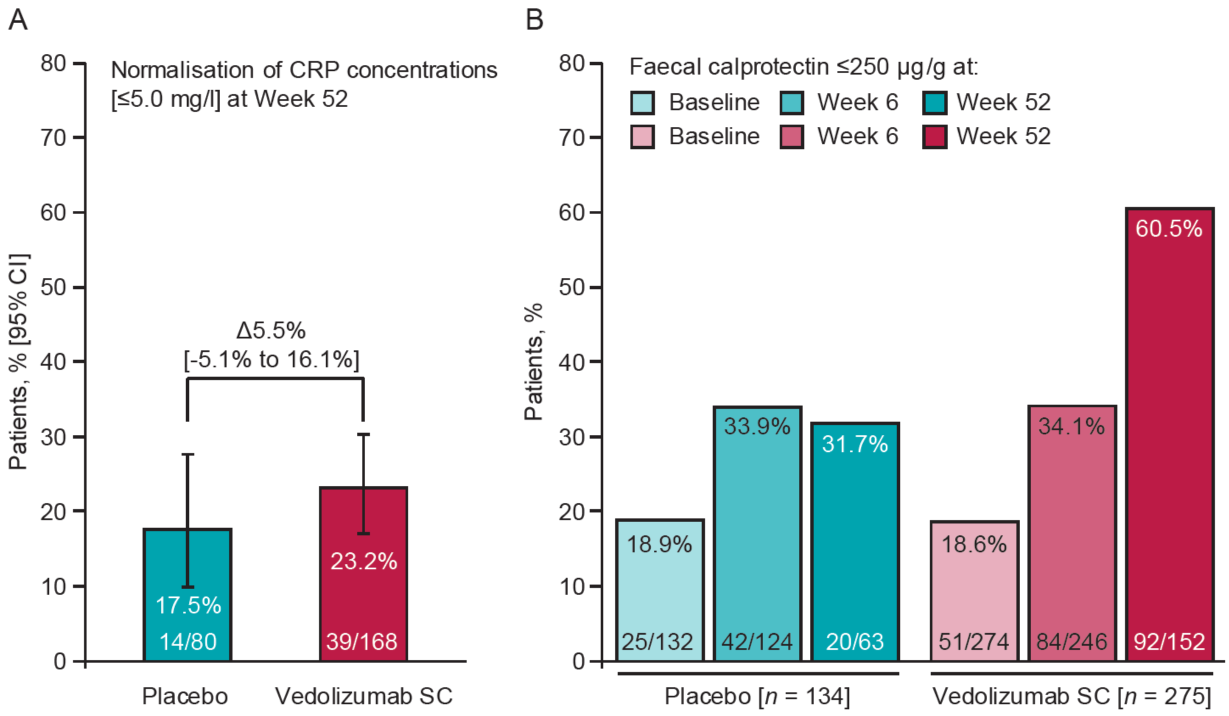

Supplementary Figure 4. Biomarker endpoints [full analysis set]. [A] Normalisation of CRP [defined as CRP >5 mg/l at baseline and ≤5 mg/l at Week 52]. [B] Patients with normal faecal calprotectin [defined as ≤250 µg/g] over time at study visits. CI, confidence interval; CRP, C-reactive protein; SC, subcutaneous.

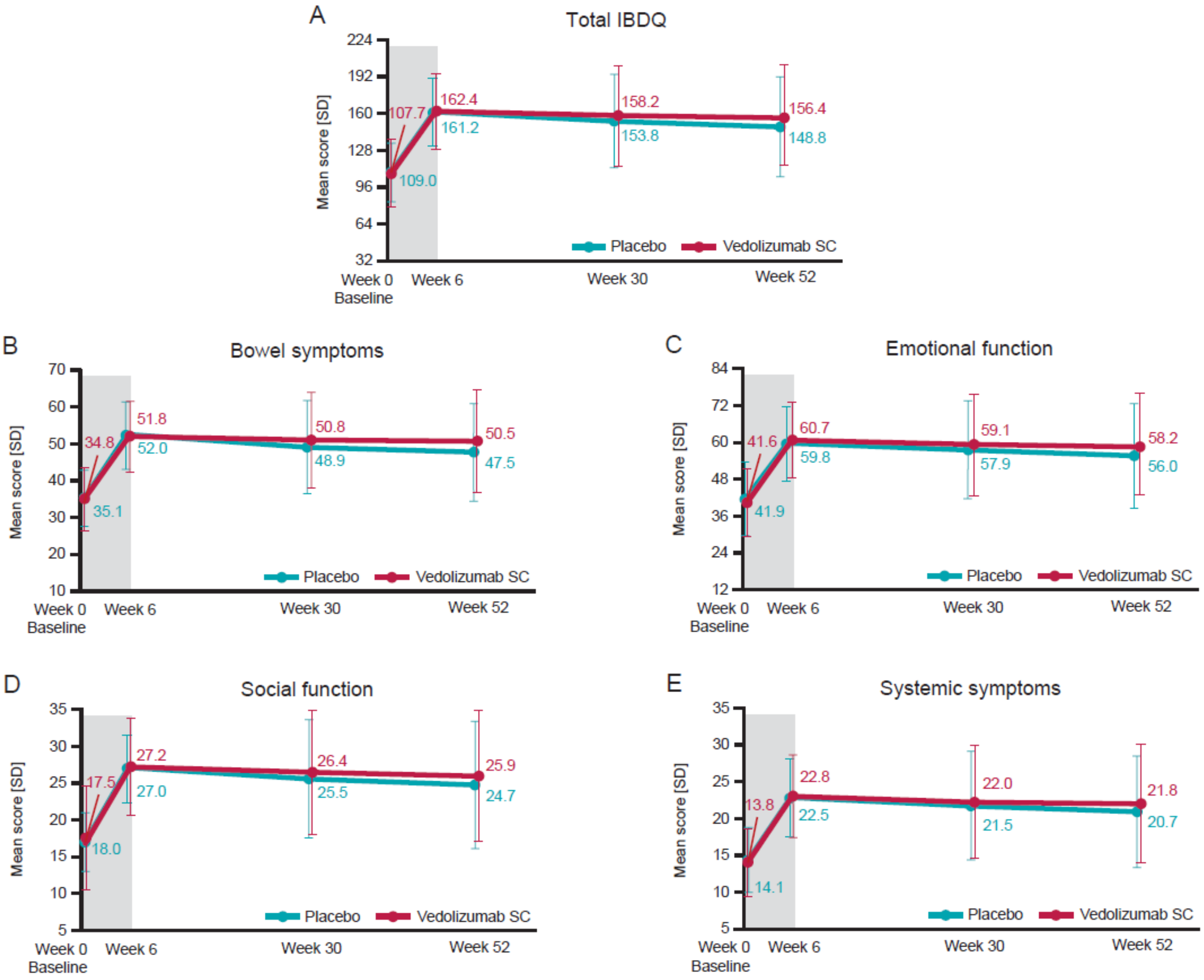

Supplementary Figure 5. [A] Total IBDQ score and [B–E] IBDQ subscores by study visit. Grey shading denotes open-label vedolizumab intravenous induction treatment. IBDQ, Inflammatory Bowel Disease Questionnaire; SC, subcutaneous; SD, standard deviation.

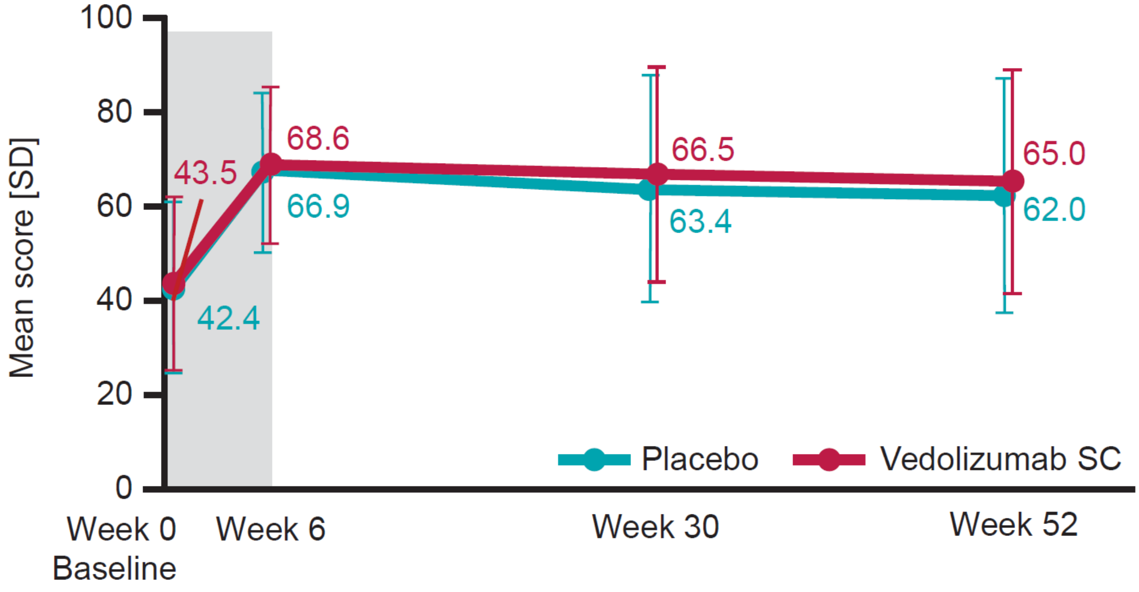

Supplementary Figure 6. EuroQol 5-Dimensions visual analogue scale scores by study visit. Grey shading denotes open-label vedolizumab intravenous induction treatment. SC, subcutaneous; SD, standard deviation.

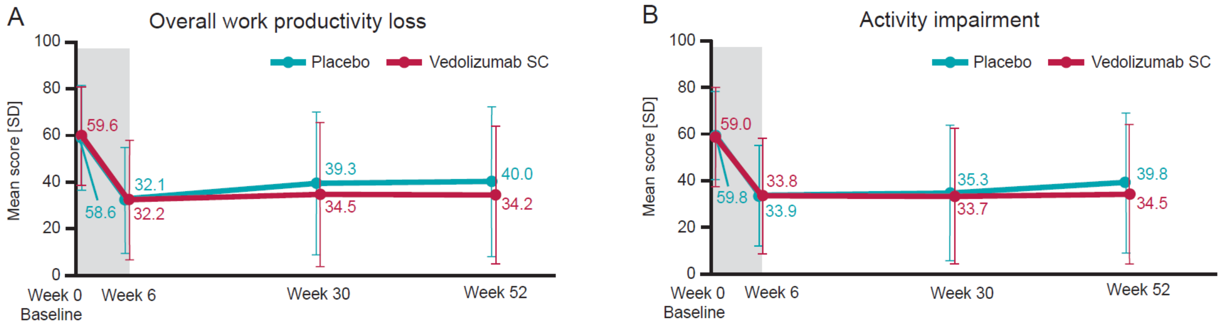

Supplementary Figure 7. The Work Productivity and Activity Impairment–Crohn's Disease scale [A] overall work productivity loss and [B] activity impairment scores by study visit. Grey shading denotes open-label vedolizumab intravenous induction treatment. SC, subcutaneous; SD, standard deviation.

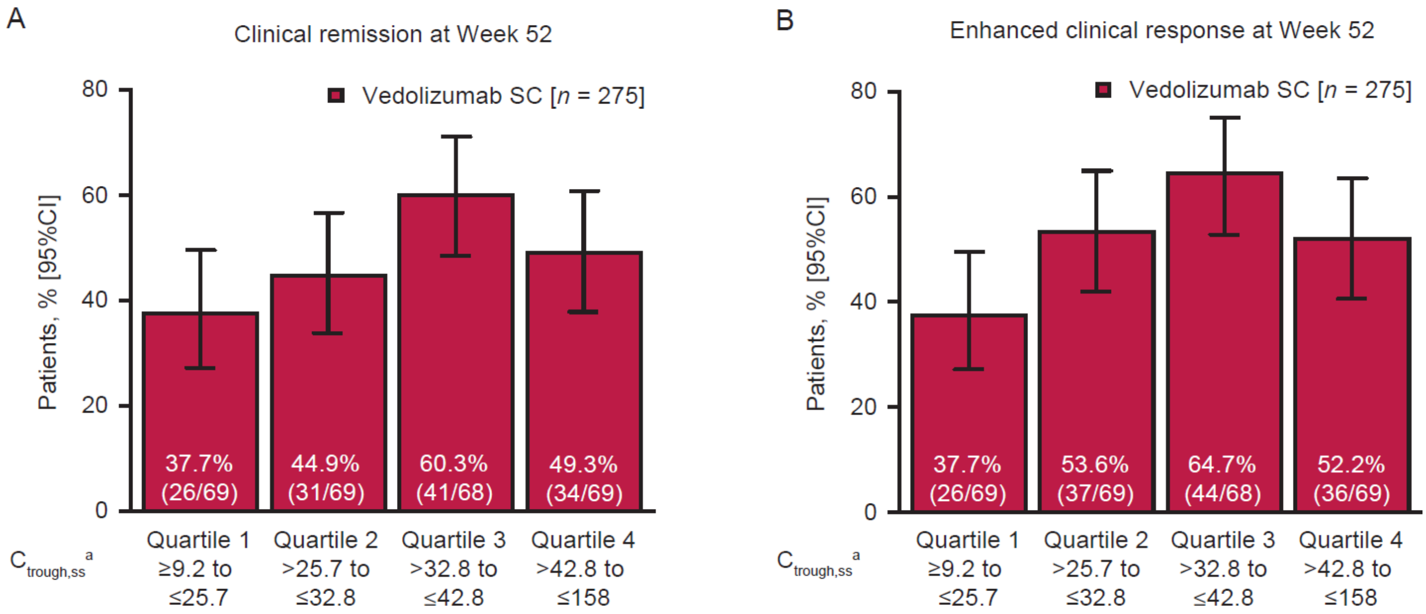

Supplementary Figure 8. Exposure-response relationship for [A] clinical remission at Week 52 and [B] enhanced clinical response at Week 52 by trough concentration quartiles.[1]

[a]Vedolizumab predicted steady-state trough concentrations ($C_{trough,ss}$) based on the population pharmacokinetics model were grouped by quartiles.

Supplementary Table 1. Study inclusion and exclusion criteria.

Inclusion
• Adults aged 18–80 years old<br>• Diagnosis of CD established ≥3 months before screening by clinical and endoscopic evidence and corroborated by a histopathology report. Cases of CD established ≥6 months before screening for which a histopathology report was not available were to be considered on a case-by-case basis, based on the weight of evidence supporting the diagnosis and excluding other potential diagnoses<br>• Moderately to severely active CD [CD Activity Index score of 220–450] within 7 days before the first dose of study drug and one of the following:<br>o C-reactive protein >2.87 mg/l during the screening period<br>o Ileocolonoscopy with a minimum of three non-anastomotic ulcerations [each >0.5 cm in diameter] or 10 aphthous ulcerations [involving a minimum of 10 contiguous centimetres of intestine] consistent with CD, within 4 months before screening<br>o Faecal calprotectin >250 µg/g stool during the screening period in conjunction with computed tomography enterography, magnetic resonance enterography, contrast-enhanced small bowel radiography, or wireless capsule endoscopy revealing CD ulcerations [aphthae not sufficient], within 4 months before screening<br>• Involvement of the ileum and/or colon, at a minimum

- Patients with >8 years duration of extensive colitis or pancolitis or left-sided colitis of >12 years duration must have documented surveillance endoscopy performed within 12 months of screening
- Cancer surveillance must be up to date in patients with known risk factors, or conducted during screening
- Inadequate response to, loss of response to, or intolerance of at least one of the following: immunomodulators, corticosteroids, or anti-tumour necrosis factor therapies

Exclusion

Gastrointestinal exclusion criteria

- Abdominal abscess, extensive colonic resection, subtotal or total colectomy
- History of more than three small bowel resections or diagnosis of short bowel syndrome
- Received tube feeding, defined formula diets, or parenteral alimentation within 28 days before the administration of the first dose of study drug
- Previous ileostomy, colostomy, or known fixed symptomatic stenosis of the intestine
- Receipt of any investigational or approved biologic/biosimilar within 60 days or 5 half-lives of screening, or receipt of any non-permitted investigational or approved non-biologic therapies within 30 days or 5 half-lives of screening
    - Oral 5-ASA probiotics and antibiotics were permitted if doses were stable for 2 weeks before the first dose of the study and remained stable throughout the study. Anti-diarrhoeals were permitted. Azathioprine, 6-mercaptopurine, or methotrexate could be continued if the patient’s dose had been stable for 8 weeks before the start of the study

- Topical [rectal] treatment with 5-ASAs or corticosteroid enemas/suppositories within 2 weeks of the administration of the first dose of study drug
- Requirement or anticipated requirement for surgical intervention for CD during the study
- History or evidence of adenomatous colonic polyps that had not been removed or colonic mucosal dysplasia
- Suspected or confirmed diagnosis of ulcerative colitis, indeterminate colitis, ischaemic colitis, radiation colitis, diverticular disease associated with colitis, or microscopic colitis

Infectious disease exclusion criteria

- Evidence of an active infection during the screening period
- Evidence of, or treatment for, *Clostridium difficile* infection or other intestinal pathogen within 28 days before the first dose of study drug
- Chronic HBV infection or chronic hepatitis C virus infection
    - HBV-immune patients may have been included
- Active or latent tuberculosis
- Any identified congenital or acquired immunodeficiency [eg, common variable immunodeficiency, HIV infection, organ transplantation]
- Receipt of any live vaccinations within 30 days before screening

- Clinically significant infection [eg, pneumonia, pyelonephritis] within 30 days before screening, or ongoing chronic infection

General exclusion criteria

- Previous exposure to approved or investigational anti-integrin antibodies (eg, natalizumab, efalizumab, etrolizumab, abrilumab [AMG 181]), anti-mucosal addressin cell adhesion molecule-1 antibodies, or rituximab
- Previous exposure to vedolizumab
- Hypersensitivity or allergies to any of the vedolizumab excipients
- Any unstable or uncontrolled cardiovascular, pulmonary, hepatic, renal, gastrointestinal, genitourinary, haematological, coagulation, immunological, endocrine/metabolic, or other medical disorder that, in the opinion of the investigator, would confound the study results or compromise patient safety
- Any surgical procedure requiring general anaesthesia within 30 days before screening or plan to undergo major surgery during the study period
- Any history of malignancy, except for the following: [1] adequately treated non-metastatic basal cell skin cancer; [2] squamous cell skin cancer that had been adequately treated and that had not recurred for ≥1 year before screening; and [3] history of cervical carcinoma in situ that had been adequately treated and that had not recurred for ≥3 years before screening. Patients with remote history of malignancy [eg, >10 years since completion of curative therapy without recurrence] were to be considered on a case-by-case basis based on the nature of the malignancy and the therapy received

- History of any major neurological disorders, including stroke, multiple sclerosis, brain tumour, or neurodegenerative disease
- Positive progressive multifocal leukoencephalopathy subjective symptom checklist at screening [or before the administration of the first dose of study drug at Week 0]
- Any of the following laboratory abnormalities during the screening period:
  - Haemoglobin level <8 g/dL
  - White blood cell count $<3 \times 10^9$/l
  - Lymphocyte count $<0.5 \times 10^9$/l
  - Platelet count $<100 \times 10^9$/l or $>1200 \times 10^9$/l
  - Alanine aminotransferase or aspartate aminotransferase >3 × ULN
  - Alkaline phosphatase >3 × ULN
  - Serum creatinine >2 × ULN
- History of drug abuse [defined as any illicit drug use] or a history of alcohol abuse within 1 year before screening
- Active psychiatric problem that, in the investigator's opinion, may have interfered with compliance with study procedures
- Patient or caregiver was unable to attend all the study visits or comply with study procedures
- Unwilling or unable to self-inject, or did not have a caregiver [defined as a legal adult] to inject the study medication
- Lactation or pregnancy during the screening period or a positive urine pregnancy test at Week 0, before study drug administration

- Intention to reproduce before, during, or within 18 weeks after participating in this study
- Immediate family member, study site employee, or in a dependent relationship with a study site employee who was involved in conduct of this study [eg, spouse, parent, child, sibling], or may have consented under duress

5-ASA, 5-aminosalicylates; CD, Crohn’s disease; HBV, hepatitis B virus; ULN, upper limit of normal.

Supplementary Table 2. Primary and secondary efficacy endpoints [per protocol and post hoc sensitivity analysis sets].

Week 52 endpoint	Placebo, *n*/*N* [%]	Vedolizumab SC, *n*/*N* [%]	Nominal *p*-value	Treatment difference, % [95% CI]
Per protocol set, N	117	248		
Primary endpoint				
Clinical remission[a]	39/117 [33.3]	126/248 [50.8]	0.002	17.6 [7.1 to 28.1]
Secondary efficacy endpoints				
Enhanced clinical response[b]	49/117 [41.9]	135/248 [54.4]	0.021	12.9 [2.1 to 23.7]
Corticosteroid-free clinical remission[c]	6/37 [16.2]	43/89 [48.3]	<0.001	32.1 [16.2 to 47.9]
Clinical remission at Week 52 in anti-TNF-naïve patients	25/58 [43.1]	51/99 [51.5]	0.412*	7.0 [-9.6 to 23.6]
Post hoc sensitivity analysis[d], N	130	261		
Primary endpoint				
Clinical remission[a]	45/130 [34.6]	126/261 [48.3]	0.010	13.6 [3.5 to 23.7]
Secondary efficacy endpoints, n				
Enhanced clinical response[b]	60/130 [46.2]	137/261 [52.5]	0.234	6.3 [-4.1 to 16.8]
Corticosteroid-free clinical remission[c]	8/42 [19.0]	38/88 [43.2]	0.007	24.4 [8.7 to 40.2]

Clinical remission at Week 52 in anti-TNF-naïve patients	26/61 [42.6]	51/102 [50.0]	0.479	5.8 [-10.4 to 22.1]

The treatment difference, the associated 95% CIs, and *p*-values were obtained using the Cochran-Mantel-Haenszel test stratified by randomisation stratum. All patients with missing data for determination of endpoint status were categorised as non-remitters. *Nominal *p*-values that cannot be considered for statistical significance.

anti-TNF, anti-tumour necrosis factor; CI, confidence interval; SC, subcutaneous.

[a]Clinical remission was defined as Crohn's Disease Activity Index score ≤150 at Week 52.

[b]Enhanced clinical response was defined as a ≥100-point decrease in Crohn's Disease Activity Index score from baseline.

[c]Corticosteroid-free clinical remission was defined as patients using oral corticosteroids at baseline who had discontinued oral corticosteroids and were in clinical remission at Week 52.

[d]Excluding 18 patients [4 in placebo arm and 14 in vedolizumab SC arm] who were randomised but who did not meet the Crohn's Disease Activity Index threshold of change for clinical response.

Supplementary Table 3. Observed faecal calprotectin concentrations by study visit [full analysis set].

Study visit	Placebo [*n* = 134]	Vedolizumab SC [*n* = 275]
Baseline[a]		
n	132	274
≤250 µg/g, *n* [%]	25 [18.9]	51 [18.6]
>250 to ≤500 µg/g, *n* [%]	22 [16.7]	49 [17.9]
>500 µg/g, *n* [%]	85 [64.4]	174 [63.5]
Week 6		
n	124	246
≤250 µg/g, *n* [%]	42 [33.9]	84 [34.1]
>250 to ≤500 µg/g, *n* [%]	17 [13.7]	40 [16.3]
>500 µg/g, *n* [%]	65 [52.4]	122 [49.6]
Week 30		
n	87	175
≤250 µg/g, *n* [%]	32 [36.8]	89 [50.9]
>250 to ≤500 µg/g, *n* [%]	9 [10.3]	21 [12.0]
>500 µg/g, *n* [%]	46 [52.9]	65 [37.1]
Week 52		
n	63	152
≤250 µg/g, *n* [%]	20 [31.7]	92 [60.5]
>250 to ≤500 µg/g, *n* [%]	10 [15.9]	18 [11.8]
>500 µg/g, *n* [%]	33 [52.4]	42 [27.6]

SC, subcutaneous.

[a]Baseline was defined as the last non-missing measurement before or on the date of the first dose of study drug [Day 1].

Supplementary Table 4. Injection site reactions [safety analysis set[a]].

Variable, *n* [%]	Placebo [*n* = 134]	Vedolizumab SC [*n* = 275]
Patients with injection site reaction	2 [1.5]	8 [2.9][b]
Injection site erythema	0	3 [1.1]
Injection site pain	1 [0.7]	1 [0.4]
Injection site pruritus	0	2 [0.7]
Injection site reaction	0	1 [0.4]
Injection site bruising	1 [0.7]	0
Injection site rash	0	1 [0.4]
Injection site urticaria	0	1 [0.4]

SC, subcutaneous.

[a]The safety analysis set included all patients who were randomised to the maintenance phase and received at least one dose of study drug.

[b]Includes one patient with an adverse event of hypersensitivity that included oedema at the injection site, along with other symptoms.

Supplementary Table 5. Infections and infestations [safety analysis set[a]].

Variable, *n* [%]	Placebo<br>[*n* = 134]	Vedolizumab SC<br>[*n* = 275]
Patients with at least one infection	46 [34.3]	86 [31.3]
Upper respiratory tract infections	19 [14.2]	49 [17.8]
Abdominal and gastrointestinal infections	7 [5.2]	11 [4.0]
Lower respiratory tract and lung infections	4 [3.0]	11 [4.0]
Influenza viral infections	5 [3.7]	9 [3.3]
Ear infections	3 [2.2]	5 [1.8]
Herpes viral infections	6 [4.5]	4 [1.5]
Viral infections NEC	1 [0.7]	6 [2.2]
Urinary tract infections	6 [4.5]	1 [0.4]
Fungal infections NEC	1 [0.7]	4 [1.5]
Skin structures and soft tissue infections	1 [0.7]	4 [1.5]
Eye and eyelid infections	3 [2.2]	1 [0.4]
Infections NEC	1 [0.7]	3 [1.1]
Dental and oral soft tissue infections	2 [1.5]	1 [0.4]
Streptococcal infections	1 [0.7]	1 [0.4]
Bacterial infections NEC	0	1 [0.4]
Campylobacter infections	0	1 [0.4]
Clostridia infections	0	1 [0.4]
Flaviviral infections	1 [0.7]	0
Pseudomonal infections	0	1 [0.4]
Tinea infections	0	1 [0.4]

NEC, not elsewhere classified; SC, subcutaneous.

[a]The safety analysis set included all patients who were randomised to the maintenance phase and received at least one dose of study drug.

Supplementary Table 6. Clinical remission at Week 52 by overall ADA status [safety analysis set[a]].

	Placebo [*n* = 134]	Vedolizumab SC [*n* = 275]
Patients in clinical remission, *n* [%]	46 [34.3]	132 [48.0]
ADA negative[b]	33 [71.7]	130 [98.5]
ADA positive[c]	13 [28.3]	2 [1.5]
Persistently positive[d]	10 [21.7]	1 [0.8]
Neutralizing ADA[e]	8 [17.4]	2 [1.5]
Patients not in clinical remission, *n* [%]	88 [65.7]	143 [52.0]
ADA negative[b]	69 [78.4]	138 [96.5]
ADA positive[c]	19 [21.6]	5 [3.5]
Persistently positive[d]	14 [15.9]	2 [1.4]
Neutralizing ADA[e]	10 [11.4]	2 [1.4]

Clinical remission was defined as a Crohn's Disease Activity Index score ≤150. All patients with missing data for determination of endpoint status were categorised as non-remitters. Overall ADA was defined from baseline [inclusive] through Week 52.

ADA, anti-drug antibody; SC, subcutaneous.

[a]The safety analysis set included all patients who were randomised to the maintenance phase and received at least one dose of study drug.

[b]Negative ADA was defined as a negative [not confirmed positive] ADA result at all visits.

[c]Positive ADA was defined as a confirmed positive ADA result at one or more visits.

[d]Persistently positive ADA was defined as a confirmed positive ADA result at two or more consecutive visits.

[e]Positive neutralizing ADA was defined as a positive result in the neutralizing ADA assay at any visit.

Supplementary Table 7. Continued oral corticosteroid use at Week 52 among those patients on baseline corticosteroids who achieved primary and secondary clinical outcomes at Week 52 [full analysis set].

	Placebo	Vedolizumab SC
Oral corticosteroid use at baseline, N	44	95
Patients in clinical remission at Week 52, *n*	16	50
Patients with enhanced clinical response at Week 52, *n*	21	53
Anti-TNF-naïve patients in clinical remission at Week 52,[a] *n*	11	21
Oral corticosteroid use at Week 52 among baseline users with clinical outcome, *n/n* [%]		
Patients in clinical remission at Week 52, *n*	8/16 [50.0]	7/50 [14.0]
Patients with enhanced clinical response at Week 52	8/21 [38.1]	8/53 [15.1]
Anti-TNF-naïve patients in clinical remission at Week 52	7/11 [63.6]	5/21 [23.8]

Data are from post hoc analyses. Clinical remission was defined as a Crohn's Disease Activity Index score ≤150. Enhanced clinical response was defined as a ≥100-point decrease in Crohn's Disease Activity Index score from baseline [Week 0]. All patients with missing data for determination of endpoint status were categorised as non-responders.

[a]Oral corticosteroid use at baseline: placebo, N = 22; vedolizumab SC, N = 39.

anti-TNF, anti-tumour necrosis factor; SC, subcutaneous

References

Chen C, Rosario M, Polhamus D, Dirks NL, Zhang W, Sun W, Kisfalvi K, Feagan BG, Sandborn WJ, Vermeire S, D'Haens G. An Evaluation of the Exposure-Efficacy Relationship for Subcutaneous Vedolizumab Maintenance Treatment of Crohn's Disease: Pharmacokinetics Findings from VISIBLE 2. Poster presented at: 2020 European Crohn's and Colitis Organisation Congress, February 12-15, 2020, Vienna, Austria. DOP16.
